# ELF5 Suppresses Estrogen Sensitivity and Underpins the Acquisition of Antiestrogen Resistance in Luminal Breast Cancer

**DOI:** 10.1371/journal.pbio.1001461

**Published:** 2012-12-27

**Authors:** Maria Kalyuga, David Gallego-Ortega, Heather J. Lee, Daniel L. Roden, Mark J. Cowley, C. Elizabeth Caldon, Andrew Stone, Stephanie L. Allerdice, Fatima Valdes-Mora, Rosalind Launchbury, Aaron L. Statham, Nicola Armstrong, M. Chehani Alles, Adelaide Young, Andrea Egger, Wendy Au, Catherine L. Piggin, Cara J. Evans, Anita Ledger, Tilman Brummer, Samantha R. Oakes, Warren Kaplan, Julia M. W. Gee, Robert I. Nicholson, Robert L. Sutherland, Alexander Swarbrick, Matthew J. Naylor, Susan J. Clark, Jason S. Carroll, Christopher J. Ormandy

**Affiliations:** 1 Cancer Research Program and The Kinghorn Cancer Centre, Garvan Institute of Medical Research, Darlinghurst, NSW, Australia; 2 Peter Wills Bioinformatics Centre, Garvan Institute of Medical Research, Darlinghurst, NSW, Australia; 3 Cancer Research UK, Cambridge Research Institute, Cambridge, United Kingdom; 4 School of Mathematics and Statistics, and Prince of Wales Clinical School, University of New South Wales Kensington NSW, Australia; 5 University of Bath, Bath, United Kingdom; 6 St Vincent's Clinical School, St Vincent's Hospital and University of New South Wales, Darlinghurst NSW, Australia; 7 Breast Cancer (Molecular Pharmacology) Group, Cardiff School of Pharmacy and Pharmaceutical Science, Cardiff University, Cardiff, Wales, United Kingdom; 8 Discipline of Physiology & Bosch Institute, School of Medical Sciences, University of Sydney, NSW, Australia; Friedrich Miescher Institute, Switzerland

## Abstract

We have previously shown that during pregnancy the E-twenty-six (ETS) transcription factor ELF5 directs the differentiation of mammary progenitor cells toward the estrogen receptor (ER)-negative and milk producing cell lineage, raising the possibility that ELF5 may suppress the estrogen sensitivity of breast cancers. To test this we constructed inducible models of *ELF5* expression in ER positive luminal breast cancer cells and interrogated them using transcript profiling and chromatin immunoprecipitation of DNA followed by DNA sequencing (ChIP-Seq). ELF5 suppressed ER and FOXA1 expression and broadly suppressed ER-driven patterns of gene expression including sets of genes distinguishing the luminal molecular subtype. Direct transcriptional targets of ELF5, which included *FOXA1*, *EGFR*, and *MYC*, accurately classified a large cohort of breast cancers into their intrinsic molecular subtypes, predicted ER status with high precision, and defined groups with differential prognosis. Knockdown of ELF5 in basal breast cancer cell lines suppressed basal patterns of gene expression and produced a shift in molecular subtype toward the claudin-low and normal-like groups. Luminal breast cancer cells that acquired resistance to the antiestrogen Tamoxifen showed greatly elevated levels of ELF5 and its transcriptional signature, and became dependent on ELF5 for proliferation, compared to the parental cells. Thus ELF5 provides a key transcriptional determinant of breast cancer molecular subtype by suppression of estrogen sensitivity in luminal breast cancer cells and promotion of basal characteristics in basal breast cancer cells, an action that may be utilised to acquire antiestrogen resistance.

## Abstract 2

The transcription factor ELF5 is responsible for gene expression patterning underlying molecular subtypes of breast cancer and may mediate acquired resistance to anti-estrogen therapy.

## Author Summary

The molecular subtypes of breast cancer are distinguished by their intrinsic patterns of gene expression and can be used to group patients with different prognoses and treatment options. Although molecular subtyping tests are currently under evaluation, some of them are already in use to better tailor therapy for patients; however, the molecular events that are responsible for these different patterns of gene expression in breast cancer are largely undefined. The elucidation of their mechanistic basis would improve our understanding of the disease process and enhance the chances of developing better predictive and prognostic markers, new therapies, and interventions to overcome resistance to existing therapies. Here, we show that the transcription factor ELF5 is responsible for much of the patterning of gene expression that distinguishes the breast cancer subtypes. Additionally, our data suggest that ELF5 may also be involved in the development of resistance to therapies designed to stop estrogen stimulation of breast cancer. These effects of ELF5 appear to represent a partial carryover into breast cancer of its normal role in the mammary gland, where it is responsible for the development of milk-producing structures during pregnancy.

## Introduction

The molecular subtypes of breast cancer are distinguished by their intrinsic patterns of gene expression [1] that have been refined to become prognostic tests under evaluation or in use [2]. Improving our understanding of the molecular events specifying these subtypes offers the hope of new predictive and prognostic markers, development of new therapies, and interventions to overcome resistance to existing therapies. The estrogen receptor (ER)positive luminal subtypes are characterized by patterns of gene expression driven by the combined direct and indirect transcriptional influences of ER and FOXA1 [3].

ELF5, also known as ESE2 [4] is a member of the epithelium specific (ESE) subgroup of the large E-twenty-six (ETS) transcription factor family [5], found in lung, placenta, kidney, and most prominently in the breast especially during pregnancy and lactation [6]–[8]. Placentation fails in *Elf5* knockout mice [9] because de novo production of ELF5 acts with CDX2 and EOMES to specify and maintain commitment to the trophoblast cell lineage [10]. The early embryo continues to repress *Elf5* expression in association with promoter methylation [11]. In the developing mammary epithelium *Elf5* is re-expressed in a mutually exclusive pattern with ER [12]. *Elf5*−/− mice produced via tetraploid embryonic stem cell rescue [12] or conditional knockout [13] showed complete failure of mammary alveolargenesis, a developmental stage driven by prolactin and progesterone. These hormones induce *Elf5* expression and re-expression of *Elf5* in prolactin receptor knockout mammary epithelium rescued alveolargenesis [14]. Forced *ELF5* expression in nulliparous mouse mammary gland produced precocious mammary epithelial cell differentiation and milk protein production. This was associated with erosion of the mammary CD61+ progenitor cell population, and conversely, *Elf5* knockout caused accumulation of this population, establishing ELF5 as a key regulator of cell fate decisions made by this progenitor cell population [12] and explaining the developmental effects described above.

The CD61+ progenitor cell is the cell of origin for basal breast cancers [15],[16] and *Elf5* is expressed predominantly by the ER− progenitor subset [17], suggesting, together with the developmental effects of Elf5 outlined above, a role for ELF5 in determining aspects of molecular subtype of breast cancer. To examine this hypothesis we manipulated the expression of ELF5 in basal and luminal breast cancer cell lines and examined the phenotypic consequences.

## Results

### *ELF5* Expression in Breast Cancer

**Figure 1:**
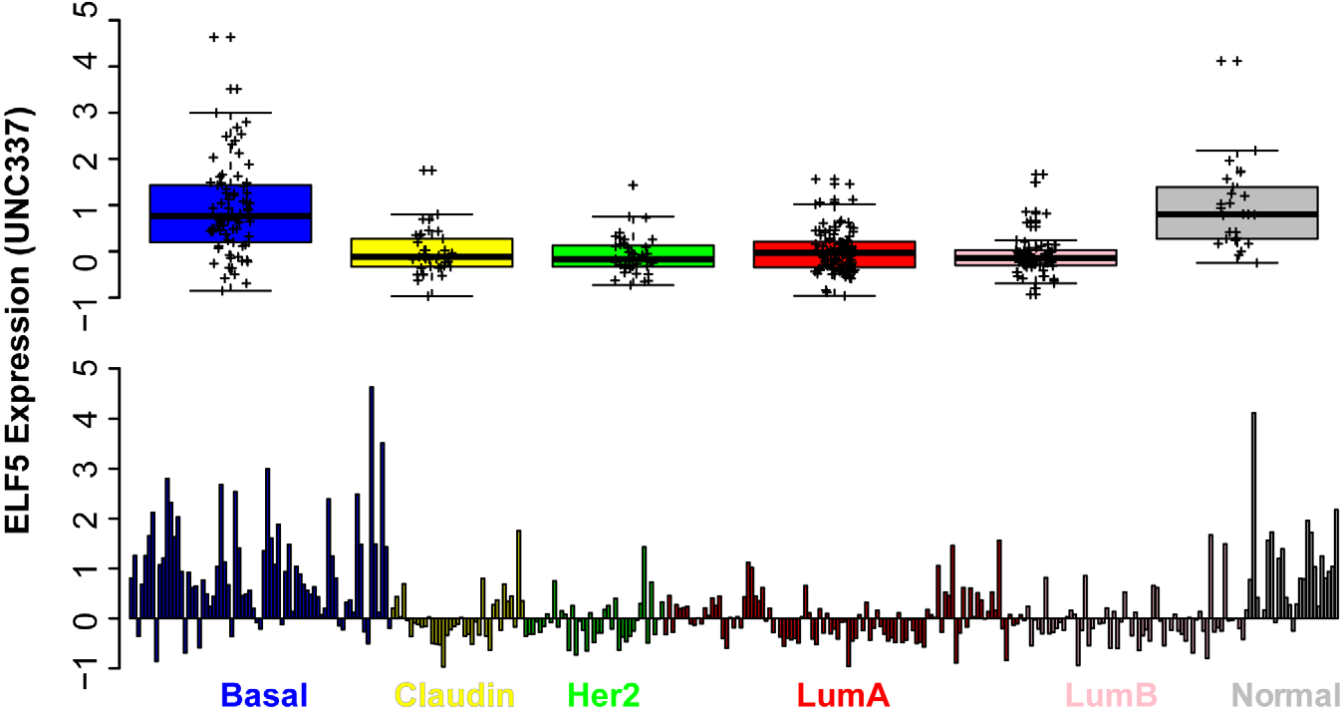
*ELF5* expression in normal breast and breast cancer. *ELF5* expression in the UNC337 breast cancer cohort according to subtype. Top panel shows combined results with statistical analysis (thick black line, median;, box, interquartile range 25%–75% of points; whiskers, 1.5× interquartile range; crosses, individual tumors). Bottom panel *ELF5* expression in all individual tumor and normal samples. Basal and normal had significantly higher expression of ELF5.

In the UNC337 breast cancer series [18] *ELF5* was expressed predominantly by the basal subtype in addition to normal breast and normal-like subtype (Figure 1), an observation confirmed in cohorts described by Pawitan [19] and Wang [20] (Figure S1). Oncomine (www.oncomine.org) revealed that *ELF5* expression was low in tumors expressing ER, progesterone receptor (PR), or ERBB2 and high in the “triple negative” subtype lacking these markers. *ELF5* expression was correlated with high grade, poor outcomes such as early recurrence, metastasis, and death, response to chemotherapy, and mutations in p53 or BrCa1, all characteristics of the basal subtypes (Figure S2). *ELF5* expression was lower in cancer compared to patient-matched and micro-dissected normal mammary epithelium (Figure S2), and a series from Sgroi and colleagues [21] found *ELF5* was one of the most consistently downregulated genes at all stages of breast carcinogenesis (Figure S1).

### An Inducible Model of *ELF5* Expression in Luminal Breast Cancer Cells

To test the ability of ELF5 to drive estrogen insensitivity we used ER+ luminal breast cell lines T47D and MCF7 to construct DOXycycline (DOX)-inducible expression models of ELF5 (Figure S3A). In humans, ELF5 is also known as ESE2 and 2 isoforms exist. The *ESE2B* isoform was expressed at 1,774- and 1,217-fold excess over the *ESE2A* isoform in MCF7 and T47D, respectively (Figure S3B). We tagged ESE2B at its C-terminus with V5 (referred to subsequently as ELF5-V5), and demonstrated that this did not alter its ability to induce the transcription of its best characterized direct transcriptional target, whey acidic protein (*Wap*) in HC11 cells (Figure S3C).

### Investigation of the Transcriptional Response to ELF5-V5

**Figure 2:**
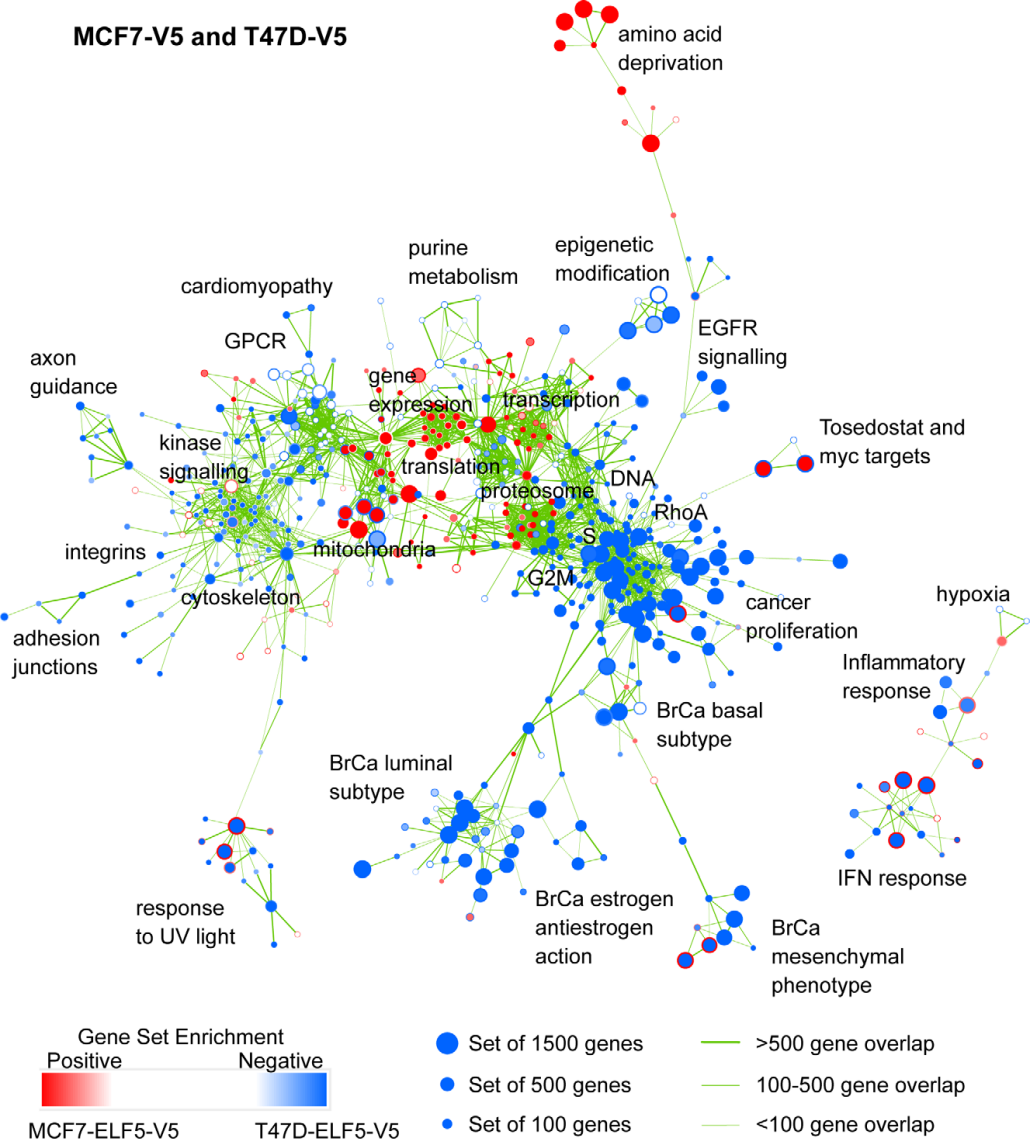
Visualization of the transcriptional functions of ELF5 in breast cancer. Affymetrix transcript profiling following induction of ELF5 in T47D and MCF7 luminal breast cancer cells, analysed by LIMMA and GSEA. Results are visualized using the enrichment map plug-in for Cytoscape. Each circular node is a gene set with diameter proportional to the number of genes. The outer node color represents the magnitude and direction of enrichment (see scale) in T47D cells, inner node color enrichment in MCF7 cells. Thickness of the edges (green lines) is proportional to the similarity of gene sets between linked nodes. The most related clusters are placed nearest to each other. The functions of prominent clusters are shown. The network can be examined in detail using the scalable PDF in Figure S4.

We interrogated our inducible models using Affymetrix arrays. Functional signatures within these expression profiles were identified by gene set enrichment analysis (GSEA) [22],[23], and were visualized using the Enrichment Map plug-in for Cytoscape [24]. The original data are available via GEO (GSE30407), and GSEA and Limma analysis from the corresponding author. Figure 2 displays the GSEA networks derived from the effects of forced *ELF*5 expression in T47D or MCF7 cells and provides a comprehensive view of the functional consequences of forced *ELF5* expression in the luminal subtype. Figure S4 provides the complete network as a fully scalable PDF allowing the identification of all nodes. Acute forced *ELF5* expression caused enhancement (positive enrichment-red nodes) of oxidative phosphorylation, translation, proteasome function, and mRNA processing. We observed suppression (negative enrichment-blue nodes) of the DNA synthetic and mitotic phases of the cell cycle, intracellular kinase signaling, cell attachment, the transmembrane transport of small molecules, transcription, and a large set of genes involved in aspects of cancer, stem cell biology, and especially the distinction of breast cancer subtypes and estrogen sensitivity. The cancer-proliferation and breast cancer subtype sub networks, the subjects of further investigation, are shown in Figures S5 and S6, and the expression of the individual genes forming the leading edges of example sets from these clusters are shown as heat maps in Figures S7, S8, S9, S10. We validated these findings using human breast cancers. Using luminal A breast cancers from the UNC337 series we produced a ranked gene list by Pearson correlation with *ELF5* expression. This approach produced an enrichment map that was very similar to that produced above (Figure 2) by forced *ELF5* expression, with cell cycle sets, cancer sets, and sets describing luminal characteristics and estrogen responsiveness prominent among the suppressed gene clusters (Figure S11), demonstrating a very similar action of endogenous ELF5 in luminal A breast cancers compared to forced ectopic expression in luminal breast cancer cells.

### Identification of ELF5 DNA Binding Sites by ChIP-Seq

**Figure 3:**
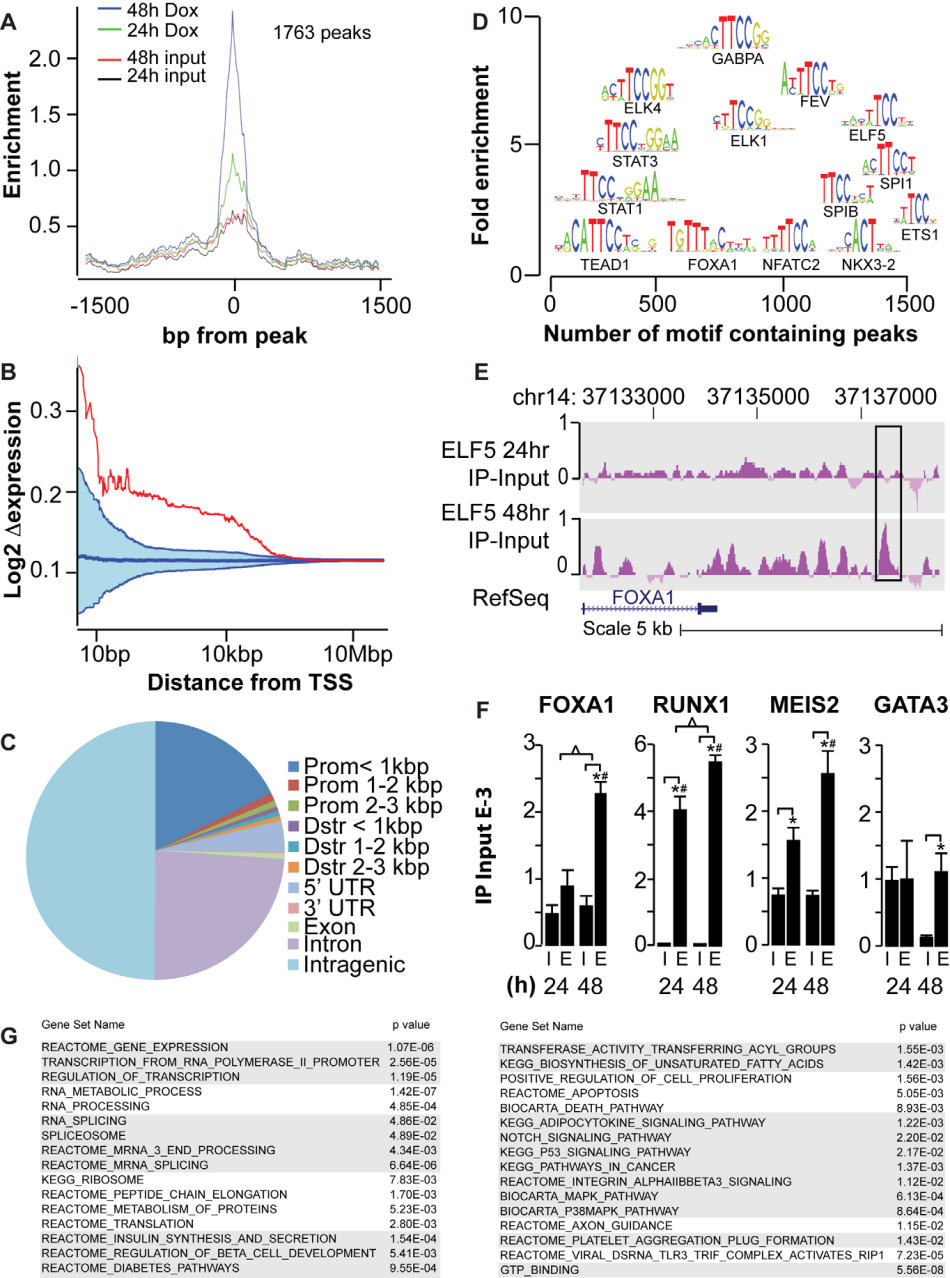
Chromatin immunoprecipitation and sequencing to identify genomic ELF5 binding sites. (A) Conservative analysis of the ChIP-Seq identified 1,763 peaks of ELF5 interaction with the genome at 48 h of DOX treatment, with a smaller number seen at 24 h. Relationship between enrichment and distance from binding peak is shown. (B) relationship between change in gene expression with 48 h of DOX treatment and distance of a peak of ELF5 DNA binding from the TSS. Blue area defines the region of random chance computed by multiple random assignments of the ChIP data to gene expression. Red line shows the actual relationship between a peak's distance from the nearest gene's TSS and its change in expression in response to ELF5-V5 induction. (C) ELF5 binding within the indicated genomic regions. (D) The number of genomic transcription factor binding motifs (TRANSFAC) found in the ChIP fragments relative to their enrichment. (E) example of an enriched peak of ELF5-V5 binding to DNA (*FOXA1* gene). (F) ChIP-qPCR validation of ELF5 binding sites to transcription factor gene promoters. I, IgG control antibody IP, E, ELF5 antibody IP, for the indicated PCR targets at 24 and 48 h; * significant (*p*<0.05) enrichment against input; # significant enrichment against RND3 control gene; Δ significant 24 h versus 48 h. (G) GSEA of curated functional sets among ELF5 ChIP targets.

We used a mixture of antibodies against V5 and ELF5 to immunoprecipitate DNA bound by ELF5-V5 in T47D cells, which we then sequenced, allowing us to map the ELF5-bound regions of the human genome and to identify the direct transcriptional targets of ELF5. Intersection of MACS and SWEMBL peak calls [25],[26] identified 1,763 common sites of ELF5 interaction in the genome at 48 h. Data are available in Table S1 or via GEO (GSE30407). DNA binding was much higher at 48 h than 24 h (Figure 3A), consistent with the observed changes in gene expression by Affymetrix arrays. Combination of the Affymetrix expression and chromatin immunoprecipitation of DNA followed by DNA sequencing (ChIP-Seq) data showed that ELF5 binding within 10 kb of a transcription start site (TSS) changed the expression level of that gene to a much greater extent than expected by chance (Figure 3B), demonstrating that ELF5 has consistent transcriptional activity via association with DNA within this range. ELF5 bound mostly to distal intragenic regions of the genome (50%) and to introns within genes (25%), but also at high frequency to promoter regions (20%) mostly within 1 kb of a TSS (18%). Downstream (Dstr) sites were seldom used. The 5′ UTR was also a frequent target of ELF5 but the 3′ UTR was infrequently targeted (Figure 3C). Transcription factor motifs (Figure 3D) contained within the DNA fragments precipitated by ELF5 were predominantly ELF5 and other ETS factor motifs; however, we also observed enrichment of sites for Stat1 and Stat3, which contain a TTCC core ets motif. We also observed very significant enrichment of sites for the *FOXA1* and *NKX3-2* transcription factors. The binding of ELF5 to the *FOXA1* promoter region is shown in Figure 3E. We validated the indicated peak on the *FOXA1* promoter, and three other target transcription factors by ChIP-qPCR (Figure 3F). *FOXA1*, *RUNX1*, *GATA3*, and *MEIS2* were validated as targets of ELF5 by comparison to input, indicating that ELF5 heads a transcriptional cascade. We searched for curated functional signatures among the ChIP targets using GSEA (Figure 3G). Many of the functional signatures observed in the ChIP data were also present in the expression data, demonstrating a direct transcriptional action of ELF5 to exert these regulatory effects.

### The Effect of ELF5 on Breast Cancer Cell Accumulation

**Figure 4:**
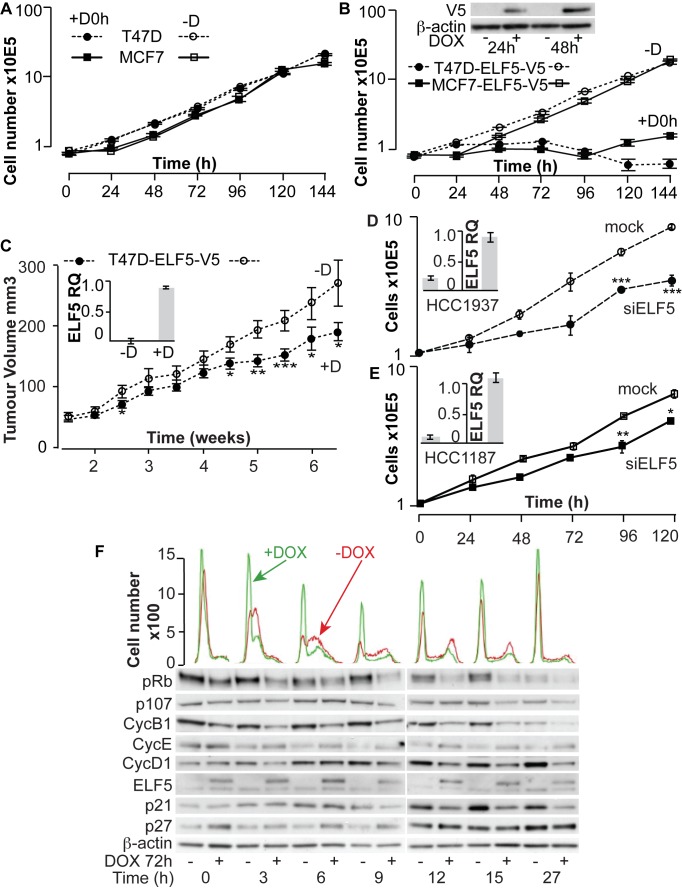
Elf5 modulates cell adhesion and proliferation of breast cancer cells. T47D (circles) and MCF7 (squares) cells were permanently transduced with DOX-inducible *ELF5-V5* or empty retroviral vectors. Pooled cells were grown with puromycin. Representative experiments are shown. Bars, standard error of the mean (SEM). (A) Control cells transduced with empty vector grown in the absence of DOX (−D, open symbols) or presence of DOX from 0 h (+D0 h, closed symbols). (B) Cells transduced with the DOX-inducible *ELF5-V5* expression vector grown in the presence of DOX (+D0 h, closed symbols) or absence of DOX (−D, open symbols). Inset Western blot of ELF5-V5 induction. (C) T47D-ELF5-V5 cells were grown as xenografts in nude mice, with (+D) or without (−D) DOX supplementation of food. Inset shows induction of *ELF5-V5* expression. (D and E) Basal breast cancer cells HCC1937 (circles, dashed lines) or HCC1187 (squares, solid lines) were transfected with siRNA against *ELF5* mRNA (siELF5, solid symbols), or were mock transfected (open symbols) before quantification of cell number with time. Insets, The degree of *ELF5* mRNA knockdown was measured by qPCR at 72 h. (F) Cells were arrested at G1-S phase by treatment with hydroxyurea, then released. Histograms show the subsequent distribution of cells within the cell cycle phases by PI staining at the indicated times post release, green +DOX, red –DOX, and Western blots show the expression of key cell cycle regulatory proteins.

We examined changes in phenotype observed in T47D-ELF5-V5 and MCF7-ELF5-V5 cells following DOX treatment. In control T47D and MCF7 cells carrying the puromycin-resistant, but otherwise empty expression vector, normal logarithmic accumulation of cells during culture continued with or without DOX (Figure 4A). In contrast, when ELF5-V5 was induced (denoted as T47D-ELF5-V5 and MCF7-ELF5-V5), cells stopped accumulating between 24 and 48 h after DOX administration (Figure 4B), regardless of the timing of induction (Figure S12A). The 4-fold induction of ELF5-V5 expression by DOX in T47D cells was similar to that produced for endogenous ELF5 with R5020, a synthetic progestin (Figure S12A, inset), demonstrating that this model produces physiological increases of ELF5 expression. The effect was also reversible (Figure S12B). Investigation of anchorage-independent growth in soft agar showed that induction of ELF5-V5 produced fewer colonies (Figure S12C). Xenografts of T47D-ELF5-V5 cells in nude mice grew at a slower rate when mice received DOX (Figure 4C).

Knockdown of ELF5 expression by more than 80% had a small effect on total cell accumulation in T47D or MCF7 cells (Figure S12D). We knocked down ELF5 in two basal breast cancer cell lines and observed a significant and sustained reduction in cell accumulation rate (Figure 4D and 4E), which was not seen in luminal cells (Figure S12D). This observation clearly demonstrates a subtype-specific role of ELF5 in breast cancer cells.

Cells can fail to accumulate in culture via two main mechanisms, by reduced rates of cell division or by the loss of cells through detachment and apoptosis. We investigated these possibilities.

We examined cell proliferation. Labeling of cells with BrdU and propidium iodide showed that induction of ELF5-V5 caused repartitioning of cells from S-phase into gap 1 of the cell cycle (G1) (Figure S13A). Western blotting showed a loss of phosphorylated forms of the pocket proteins, p130, p107, and Rb, accompanied by loss of cyclin proteins A2, B1, and D1, and accumulation of the inhibitor p21 (Figure S13B). Many of these changes also occurred at the mRNA level (Figure S13C), indicating a transcriptional basis to these changes that together suggest inhibition of proliferation by G1 arrest. To test this we arrested T47D-ELF5-V5 cells in the G1 phase of the cell cycle using hydroxyurea (HU) and then released them, by HU wash out, into cycle in the presence and absence of induction of ELF5-V5 expression (Figure 4F, corresponding flow cytometric plots in Figure S13D, and Western blot quantification in Figure S13E). Induction of ELF5-V5 reduced the percentage of cells exiting G1 into S-phase and was associated with a reduced accumulation of cyclin D1 protein and reduction in the expression of cyclin B1, demonstrating that ELF5-V5 expressing cells failed to re-enter the cell cycle from G1.

We previously formed a set of 641 genes associated with cell cycle control by a combination of genes from cell cycle–related GO ontologies [27]. This set very significantly overlapped with 125 genes repressed, and 42 genes induced, by *ELF5* expression (Figure S14A), and of these 55 were ELF5 ChIP targets (Figure S14A, Figure S14B heat maps) indicating a direct transcriptional influence of ELF5 on proliferation. Upregulated ELF5 ChIP targets were characterized by the presence of tumor suppressor genes while downregulated genes were enriched in genes controlling cell proliferation. Upregulated genes included RB1CC1 (promotes RB1 expression), TBRG1 (promotes G1 arrest via CDKN2A), IRF1 (initiates interferon response), COPS2 (p53 stabilizer), CHFR (prevents passage into mitosis), DAB2 (lost in ovarian cancer), and RAD50 (DNA damage checkpoint). Also in this group are DDIT3 (promotes apoptosis due to endoplasmic reticulum stress) and ERBB2IP (disrupts RAF/RAS signaling). ELF5 ChIP targets repressed by elevated ELF5 were characterized by genes required for mitosis, such as GTSE1 (microtubule rearrangement), KIF11 (spindle formation), FBXO3 (anaphase promoting complex), KNTC1 (mitotic check point), PPP1CC (PTW/PP1 complex member), and PMF1 (MIS12 complex chromosome alignment). Other proproliferative ELF5 ChIP targets that were downregulated include EGFR and IGF1R (potent mammary mitogen receptors), MAPK13 (downstream signaling molecule), c-MYC (key regulator of proliferation), KLF10 (transcriptional repressor of proliferation), and NME1 and SLC29A2 (required for nucleotide synthesis). This 55-gene signature is significantly enriched in many breast cancer series (Figure S14C) and showed differential expression between ER+ and ER− cancers (Figure S14B, right-hand heat map). Interestingly the mitogenic genes that are repressed by forced ELF5 expression in ER+ T47D cells are generally highly expressed in ER− cancers (Figure S14B), showing again that ELF5 has a subtype-dependent role in cell proliferation and may contribute to the proliferative drive in ER− cancers.

**Figure 5:**
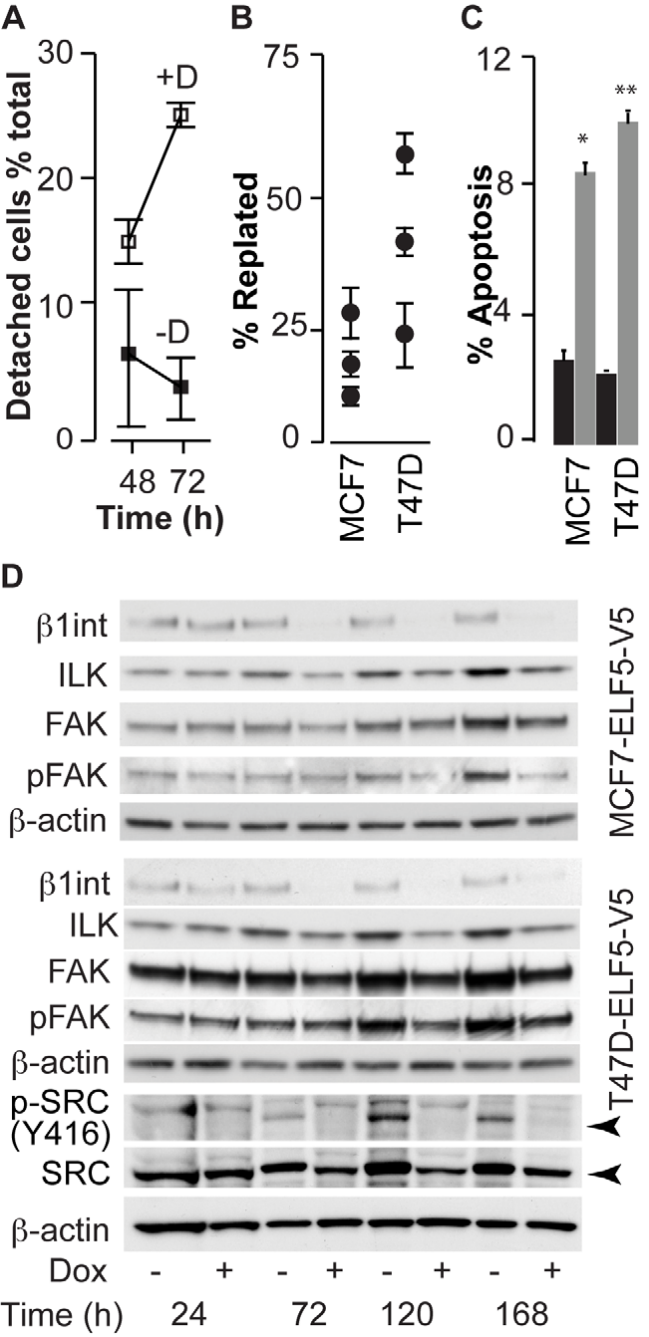
Elf5 modulates the adhesion of breast cancer cells. (A) Quantification of detached cells in cultures treated with DOX (+D) compared to no induction (−D). (B) Ability of DOX-treated cells to replate 4 h after trypsin destruction of attachment proteins, compared to untreated cells. Data are expressed as a percentage of replated untreated cells. (C) Proportion of apoptotic cells in DOX treated (grey bars) compared to untreated (black bars) T47D-ELF5-V5 cells, measured by flow cytometry using the M30 antibody. (D) Expression and activation of key cell adhesion proteins following DOX induction of ELF5-V5 expression.

A large number of detached and floating cells were observed in cultures after 48 h of DOX treatment and became most prominent by 72 h (Figure 5A). Replating efficiency was greatly reduced, indicating that new adherence proteins could not be rapidly synthesized and deployed following their destruction with trypsin (Figure 5B). Higher rates of apoptosis, measured using flow cytometry, were observed in T47D-ELF5-V5 and MCF7-ELF5-V5 cells (Figure 5C). The levels of beta 1-integrin were much lower by 72 h (Figure 5D quantitated in Figure S12E and S12F). Its signaling partner integrin-linked kinase (ILK) also showed reduced expression. Focal adhesion kinase (FAK) levels also fell slightly but phosphorylation of FAK was much reduced from 5 d, as was SRC kinase expression and especially phosphorylation, indicating reduced signal transduction in response to the lower levels of beta1-integrin and detection of the extracellular matrix. Together these results implicate loss of extracellular integrins in the detachment of cells in response to forced ELF5 expression.

### ELF5 Modulates Estrogen Action

**Figure 6:**
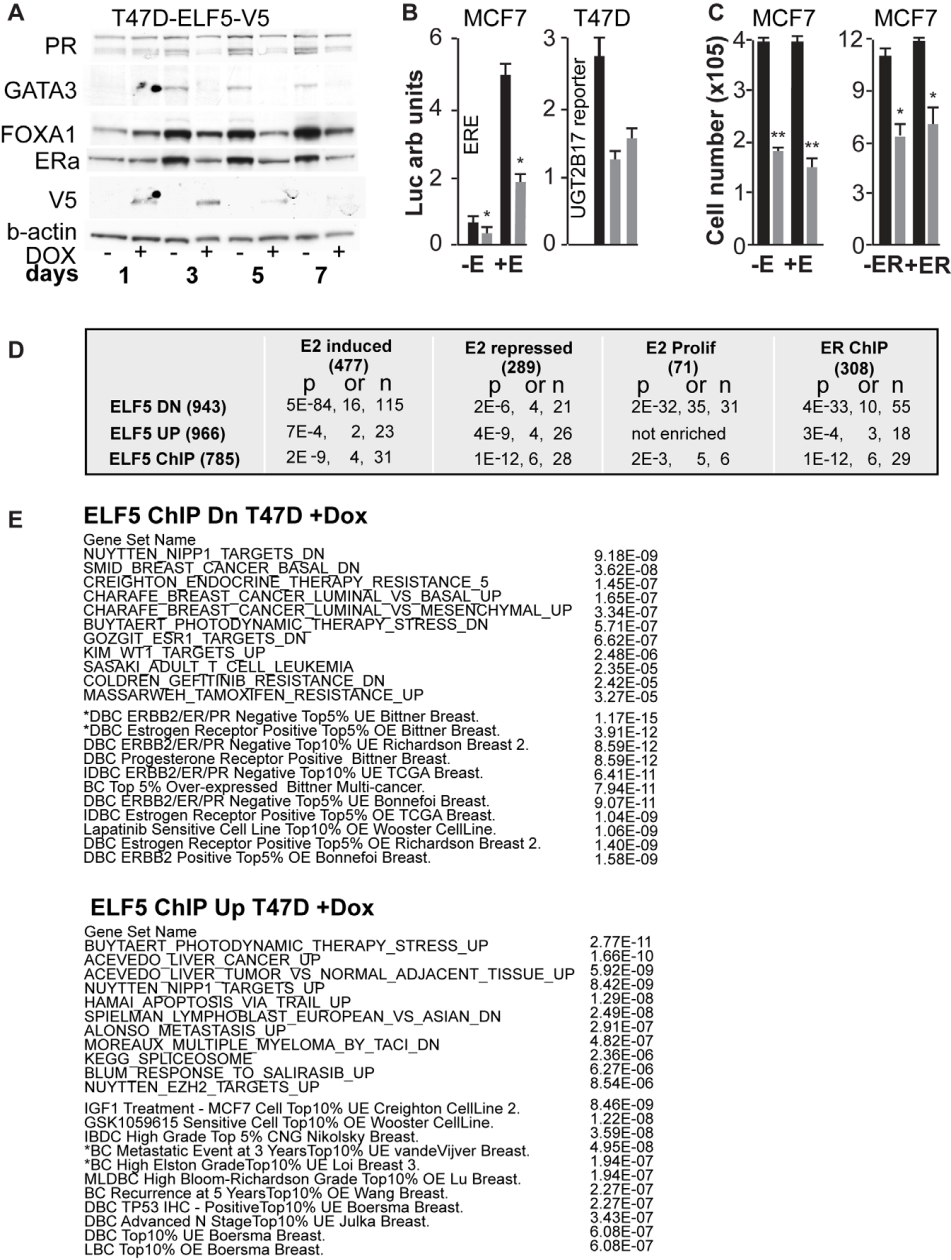
ELF5 suppresses the estrogen-sensitive phenotype. (A) Western blot showing reduced expression of key genes involved in the response to estrogens following induction of ELF5 expression. (B) Reduced transcriptional activity of reporters of *ER* and *FOXA1* (*UGT2B17* promoter) transcriptional activity following induction of ELF5 in MCF7 cells. Black bars, -DOX, grey bars +DOX 72 h for *ERE* and 24 h and 48 h for *FOXA1*. (C) Cell accumulation in MCF7-V5 cell cultures with (+E) or without (−E) 10 nM estrogen treatment, or following expression of ER (+ER) and 10 nM E in the context of induced ELF5. Black bars, -DOX; grey bars +DOX, 72 h and 144 h, respectively. (D) interaction of ELF5-regulated gene sets with estrogen-regulated gene sets. *p*-Values and odds ratios derived from hypergeometric tests. Number of genes in brackets. (E) Enrichment of gene sets in ELF5 ChIP targets either down (Dn) or Up in response to forced ELF5-V5 expression in T47D cells with DOX. P-Values for hypergeometric tests from GSEA (upper case) or Oncomine (lower case).

Among the direct transcriptional targets of ELF5 are a number with established roles in proliferation in response to estrogen, such as *FOXA1*, *MYC*, *CDK6*, *FGFR1*, and *IGF1R* (Table S1). In addition, key genes associated with the estrogen-sensitive phenotype, such as *ESR1* and estrogen-response genes, such as *GREB1* and *XBP1*, are downregulated (Figure S9). Western blotting showed that induction of ELF5-V5 expression caused falls in the levels of ER, the estrogen-induced gene progesterone receptor (PGR), pioneer factor FOXA1, and progenitor cell-regulator GATA3 (Figure 6A). The activities of ER and FOXA1 transcriptional reporters (ERE and UGT2B17, respectively) also fell (Figure 6B), demonstrating that forced ELF5 expression suppressed estrogen sensitivity. These cell lines are dependent on estrogen for proliferation, raising the possibility that forced ELF5 expression inhibited proliferation simply by reducing ER expression. We tested this possibility by forced re-expression of ER and treatment with estrogen (Figure 6C), but we did not observe any relief of the inhibition of proliferation caused by forced ELF5 expression.

We further examined the effects of induction of ELF5 on estrogen-driven gene expression by intersecting our ELF5-regulated genes with a previously defined set of estrogen-regulated genes in MCF7 cells [27]. Among a set of 477 genes showing estrogen-induced expression in MCF7 cells (Figure 6D, “E2 induced”), 115 showed loss of expression in response to ELF5-V5, an overlap with a highly significant *p*-value (*p* = 5E−84) and odds ratio (OR = 16). These genes (heat map in Figure S14D, “E2I”), contained signatures for cell cycle control and DNA replication gene sets. Furthermore when we focused on 71 estrogen-induced genes previously defined as involved in proliferation [27] (Figure 6D, “E2 Prolif”), we observed the same very significant enrichment (*p* = 2E−32), confirming the action of ELF5 to repress the expression of estrogen-induced genes involved in proliferation. The effect of ELF5 expression on 289 estrogen-repressed genes (Figure 6D, “E2 repressed” and heat map Figure S14D, “E2R”) was much less pronounced. Thus the predominant effect of forced ELF5 expression was suppression of estrogen-induced gene expression. Just 29 genes were direct transcriptional targets of both ELF5 and ER, a small fraction of the total number of genes showing changed expression, indicating that the actions of ELF5 and ER are largely executed by intermediaries, rather than direct action of ELF5 and ER at the same genomic locus.

We used hypergeometric enrichment to discover previously defined experimental signatures among the direct transcriptional targets of ELF5. Signatures indicative of estrogen action were prominent among ELF5 ChIP targets downregulated (fold change [FC]>1.5 and false discover rate [FDR]>0.25) by *ELF5* expression, including sets of genes that were ESR1 targets, involved in endocrine resistance or which distinguished the luminal from basal subtypes (Figure 6E). Among the cancer-focused sets provided by Oncomine (set names in lower case in Figure 6E) were many associated with distinction of the triple negative and ER/PR/HER2-positive subtypes. Sets among the upregulated ChIP targets included metastasis, apoptosis, and high grade. Heat maps illustrating the changed expression of the individual ELF5 ChIP targets are shown using the top-hit breast cancer series (* marked sets in Figure 6E shown as heat maps in Figure S14E). This investigation again illustrates the repression of estrogen action by induction of ELF5, but indicates that many of the poorer prognostic aspects of the ER− subtypes may be due to ELF5-induced genes. Again ELF5 appears to have subtype-specific actions.

### ELF5 Specifies Breast Cancer Subtype

We examined the ability of the direct transcriptional targets of ELF5 to predict aspects of breast cancer phenotype. A set of 164 genes was defined as ChIP targets with altered expression in response to induction of ELF5 in T47D cells. This gene set accurately predicted ER status (Figure S15A) in the Reyal breast cancer series [28]. The Confusion Matrix (Figure S15B) shows that the direct transcriptional targets of ELF5 accurately predicted intrinsic subtype, with nearly 100% of the luminal/basal distinctions correctly identified. Clustering of the NKI-295 set [29], using the direct transcriptional targets of ELF5, distinguished the intrinsic subtypes and produced a clear separation of tumor characteristics such as poor prognosis, early metastasis, early death, recurrence, survival, grade, mutation status, and marker expression, such as ER and PR (Figure S15C).

**Figure 7:**
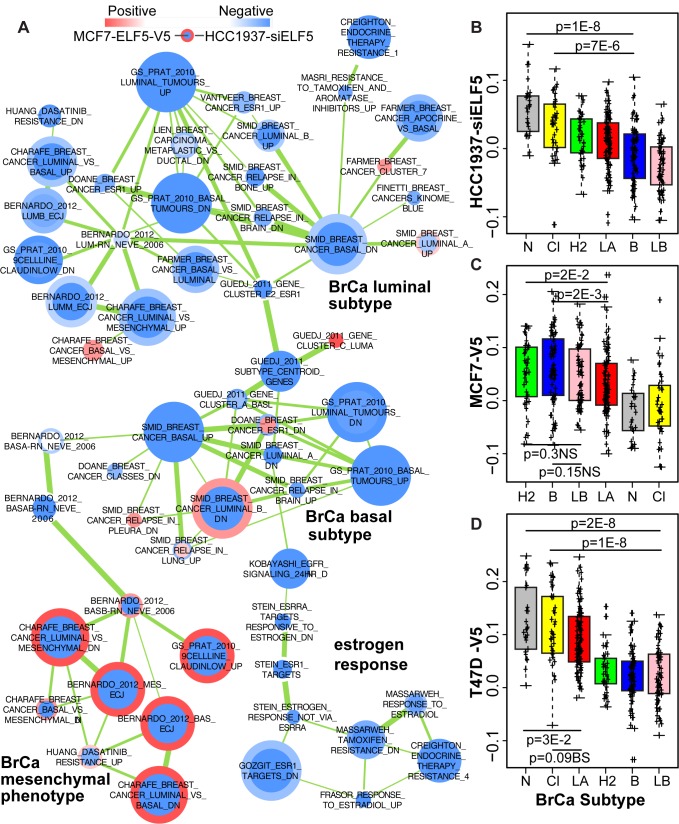
ELF5 specifies breast cancer subtype. (A) Sub network of breast cancer subtype gene sets derived from forced ELF5 expression in MCF7 luminal breast cancer cells (inner node color) and knockdown of ELF5 expression in HCC1937 basal breast cancer cells. Node size is proportional to gene set size; thicker green lines indicate greater gene set overlap. Nodes are positioned according to similarity in gene sets. Labels in bold type indicate the functional significance of the four clusters generated, label is plain type is the gene set name. The full network is shown in Figure S16. (B–D) expression signature analysis of the ELF5-induced changes in molecular subtype produced by ELF5 knockdown in HCC1937 cells (B), or forced ELF5 expression in MCF7 cells (C), or T47D cells (D). Bars show the indicated comparisons that produce the associated *p*-values. BS, borderline significance; NS, not significant.

We assessed the ability of *ELF5* expression to directly alter molecular subtype using two methods developed for this purpose, GSEA [30] and expression signature analysis [15] (Figure 7). Figure 7A shows a cytoscape network of gene sets distinguishing molecular subtype, which combines data from forced expression of *ELF5* in MCF7 luminal breast cancer cells (node center) with data from knockdown of ELF5 in HCC1937 basal breast cancer cells (outer node ring). This is a sub network of the complete cytoscape network (Figure S16) and the T47D-HCC1937 sub network (Figure S17) is almost identical. The gene sets clustered into four groups distinguishing luminal subtype, basal subtype, estrogen responsiveness, and the mesenchymal phenotype. ELF5 suppressed the mesenchymal phenotype in both luminal and basal cells, representing a subtype-independent action. In luminal cells forced *ELF5* expression suppressed the luminal subtype and estrogen-responsive phenotype. In basal cells knockdown of *ELF5* expression suppressed the basal subtype, illustrating subtype-specific actions of ELF5. Figure 7B shows the expression signature analysis. In HCC1937 cells knockdown of ELF5 produced a very significant shift in molecular subtype away from the basal subtype toward the claudin-low and normal-like subtypes, consistent with the enrichment of the mesenchymal phenotype observed by GSEA and the suppression of patterns of basal gene expression. In both luminal cell lines a shift away from the luminal subtype was observed (Figure 7C and 7D), consistent with the GSEA results. In MCF7 the shift was toward the basal and Her2+ subtypes and in T47D toward the normal-like and claudin-low subtypes.

### ELF5 Underpins the Acquisition of Antiestrogen Resistance

**Figure 8:**
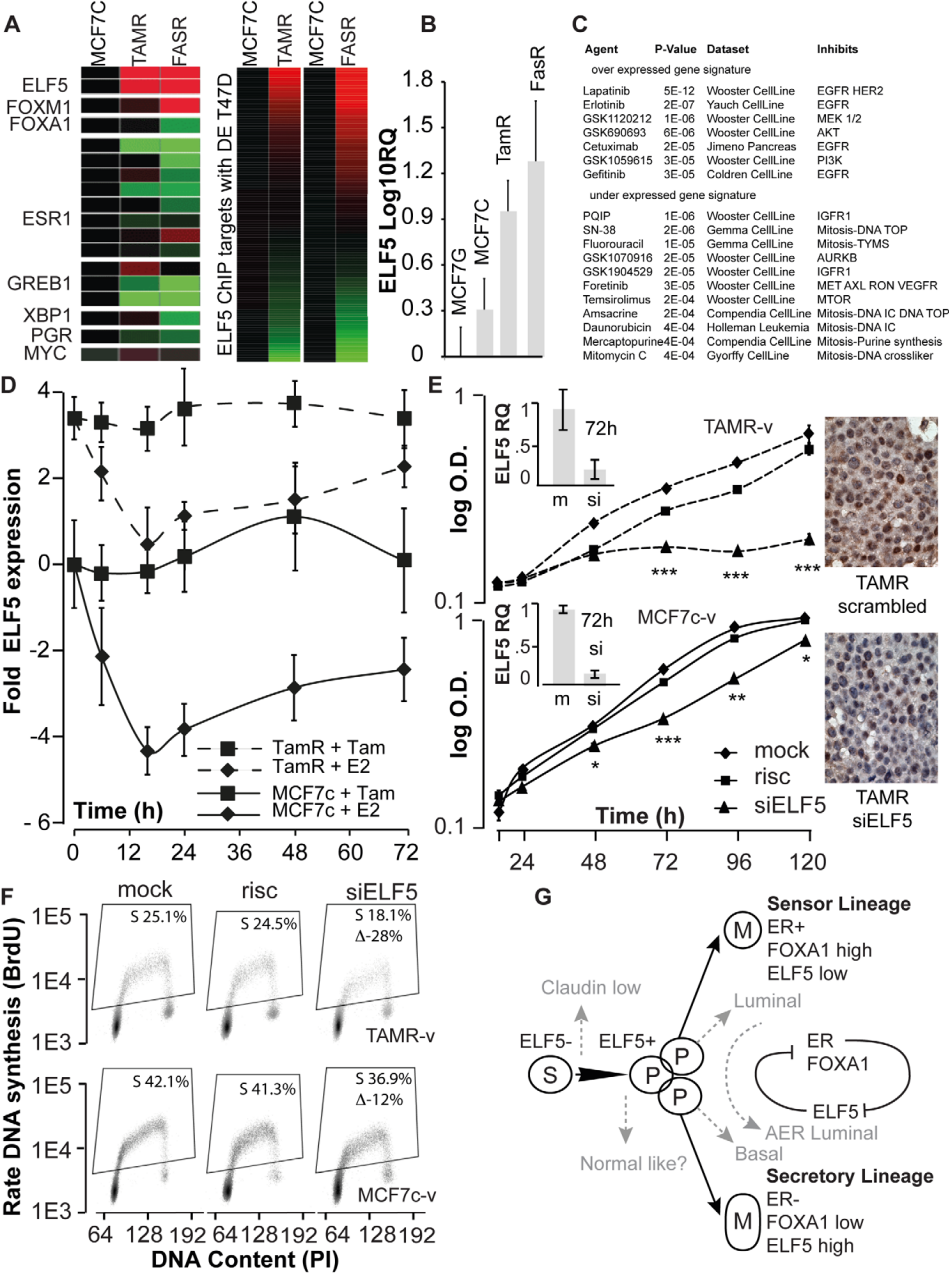
Elevated ELF5 allows escape from antiestrogen-induced proliferative arrest. (A) Affymetrix profiling of the expression (red high, green low) of key estrogen response genes in two models of antiestrogen resistance. The lines were derived from MCF7 cells from the Cardiff laboratory (MCF7C) to be resistant to TAMR or FASR. Additional two heat maps show the change in the expression of the ELF5 transcriptional signature in TAMR and FASR cells compared to parental. (B) PCR validation of the change in *ELF5* expression in TAMR and FASR cells. (C) Drug-response signatures from Oncomine enriched among genes implicated in antiestrogen resistance that are ELF5 ChIP targets. (D) Eduction in *ELF5* expression by estrogen treatment in MCF7C and TAMR cells. (E) Effects of ELF5 knockdown in TAMR cells on the expansion in cell number. Insets, demonstration of ELF5 knockdown by qPCR and IHC compared to both risc-free and scrambled siRNA controls. (F) BrdU labeling shows ELF5 knockdown inhibits cell proliferation in TAMR cells to a much greater extent than seen in the parental cells. (G) Model of ELF5 action in normal development and breast cancer, see the Discussion for details.

Expression studies and ChIP-Seq (Figures 2, 6E, and 7) showed that ELF5 transcriptionally regulated a number of genes involved in resistance to antiestrogens. We examined *ELF5* expression in Tamoxifen (TAMR)- [31] or Faslodex (FASR)- [32] resistant cells, derived from MCF7 cells in Cardiff (MCF7C). Greatly elevated levels of *ELF5* mRNA were observed (Figure 8A) compared to their parental MCF7C cells, accompanied by loss of expression of key estrogen response genes. The 164-gene ELF5 transcriptional signature used to classify subtype in Figure 6 showed a response in TAMR cells (22 genes significantly suppressed, 24 significantly induced) and FASR cells (34 genes significantly suppressed, 46 significantly induced). qPCR confirmed the elevation of *ELF5* mRNA in TAMR and FASR cells compared to the parental MCF7 (MCF7C) and MCF7 from the Garvan Institute (MCF7G) used elsewhere in this study (Figure 8B). When we intersected the genes from the antiestrogen sets enriched in the expression data in response to forced *ELF5* expression (Figure 2A) with ELF5 ChIP targets, and asked using Oncomine what drug treatments produced similar profiles, we found a predominance of signatures resembling those resulting from inhibition of EGFR (a key pathway driving Tamoxifen resistance in TAMRs [31]) among overexpressed genes, and signatures indicative of the IGFR1 pathway and other kinases, or mitosis-disrupting agents, among under expressed genes (Figure 8C). Both these pathways have been implicated in the development of resistance to antiestrogens. Treatment with estrogen greatly reduced *ELF5* expression in MCF7C cells and this effect was blunted in the TAMR cells (Figure 8D). Knockdown of ELF5 in TAMR cells completely stopped cell accumulation while in the parental MCF7C little effect on the rate of increase in cell number was seen (Figure 8E). ELF5 IHC on TAMR cell pellets confirmed the knockdown. Measurements of S-phase showed that 25% of TAMR cells and 42% of MCF7C cells were in S-phase of the cell cycle during log-phase growth, consistent with the known characteristics of these lines. Knockdown of ELF5 reduced TAMR S-phase by 28%, and MCF7C S-phase by 12% (Figure 8F), consistent with the observed effects on cell number. These observations demonstrate that elevation of *ELF5* expression and greatly increased reliance upon it for cell proliferation is a key event in the acquisition of Tamoxifen insensitivity.

Taken together, the results in this study show that ELF5 is involved in the proliferation of breast cancer cells in culture, that ELF5 suppressed the estrogen-responsive phenotype in luminal breast cancer cells, and induced aspects of the basal phenotype in basal breast cancer cells. In both subtypes ELF5 suppressed the mesenchymal phenotype. ELF5 specified patterns of gene expression that distinguished the breast cancer subtypes. Significantly for clinical management of breast cancer, elevation of ELF5 is a mechanism by which MCF7 cells can become insensitive to antiestrogen treatment.

## Discussion

In this report we show that ELF5 exerts wide transcriptional effects with functional outcomes on cell proliferation, adhesion, the molecular determinants of breast cancer subtype and phenotype, and acquired resistance to Tamoxifen. These outcomes are aspects of a general specification of an estrogen-insensitive cell fate exerted through modulation of ER, FOXA1, and other transcriptional regulators in luminal cells, and the induction of basal characteristics in basal cells.

Two key factors in determining the luminal phenotype are FOXA1 and ER. Recent findings show that ER-chromatin binding and the resulting transcriptional response to estrogens are dependent on FOXA1 expression and its action as a pioneer factor for chromatin-ER binding [3]. We show that forced expression of *ELF5* in the luminal context directly represses FOXA1 expression and transcriptional activity. We also show that ER expression falls, as does the expression of many estrogen-responsive genes and ER-driven transcription. This mechanism allows ELF5 to suppress key aspects of the luminal phenotype, demonstrated by GSEA and by expression signature analysis. The resulting loss of proliferation is likely to result from multiple mechanisms. Although the proliferation of MCF7 and T47D cells is stimulated by estrogen-driven signaling and transcription, the loss of ER signaling was not the sole cause of proliferative arrest as forced ER re-expression did not effect a rescue (Figure 6C). Rather, ELF5 may act directly to regulate genes controlling cell proliferation (Figure S14). In addition the loss of beta1-integrin was observed. Beta1-integrin acts in mammary cells via FAK and src to induce p21 and cell cycle arrest [33], a mechanism that is also apparent in our data (Figures 5D and S13C). There are additional large changes in the expression of many other key signaling molecules (Figures 2 and S4) that may also act to suppress proliferation, including EGFR and IGFR signaling. Finally the changes in metabolic activity, including protein and RNA synthesis and oxidative phosphorylation are also likely to impinge on cell proliferation.

Loss of ELF5 expression in the basal subtype HCC1937 cells resulted in reduced cell proliferation, loss of basal patterns of gene expression, and a shift toward the mesenchymal phenotype, demonstrated by the GSEA results and the expression signature analysis. These results show that ELF5 specifies key characteristics of the basal phenotype, but also prevents the expression of the mesenchymal phenotype, as it does in the luminal context. Thus we can discern both subtype-dependent and -independent effects of ELF5. The subtype-dependent effects are a likely product of the differentiation state of the cell and the fate decisions made during differentiation produced by interactions of the ELF5 regulatory transcriptional network with other transcriptional regulatory networks present in the cell, while the independent effects are likely to result from direct transcriptional actions that lack these interactions and are thus subtype independent.

We propose that the effects of ELF5 in the breast cancer cell lines represent a carry over of the normal developmental role of ELF5 into breast cancer (Figure 8G). The primary developmental target for ELF5 is the mammary progenitor cell population (P), produced from the stem cell population (S), and which exhibits aspects of the two cell lineages that it produces (drawn as P cell overlaps). Under the dominant influence of ELF5 a progenitor cell differentiates to become an ER− cell, and with further differentiation and hormonal stimulation ultimately produces a mature (M) alveolar cell capable of large-scale milk synthesis. By this process ELF5 establishes the secretory cell lineage [12]. It is likely that under a dominant estrogen influence the same progenitor cell differentiates to become an ER+ cell with different functions, such as regulation of the stem and progenitor hierarchy by paracrine influence. Recent findings support this mechanism [30]. It is likely that ELF5 and FOXA1 provide the key to the decision made by the progenitor cell. We hypothesize that the outcome of competing estrogen and ELF5 actions on a precancerous instance of the progenitor cell may play a significant role in determining the subtype of breast cancer that results. Additional events may occur subsequent to this decision that alter ELF5 expression and these may be involved in aspects of tumor progression, such as the acquisition of insensitivity to antiestrogens when ELF5 expression increases in the context of luminal breast cancer, or the acquisition of the mesenchymal phenotype when ELF5 expression is lost in basal breast cancer. Some tumors, such as the claudin low subtype, may be innately mesenchymal, as a result of the oncogenic transformation of late stem or early progenitor cells that do not yet express ELF5 or FOXA1 and ER. ELF5 may act to specify epithelial characteristics during the normal differentiation of the stem cell to become a progenitor cell. In this context forced ectopic expression of ELF5 may reduce the mesenchymal nature of the claudin low subtype.

We have observed that progestin-induced inhibition of T47D cell proliferation is accompanied by an increase in ELF5 expression, and that this in turn acts to oppose the inhibitory action of progestins on cell cycle regulation [34]. A similar effect may protect some cells from complete growth inhibition by Tamoxifen, and prime them for later ELF5-driven escape of antiestrogen therapy. Further increases in ELF5 expression provide both an alternate source of proliferative signals and further suppression of sensitivity to ER-mediated signals, producing a mechanism allowing TAMR cells to escape TAM-induced growth inhibition. In this scenario the ELF5 ChIP target c-MYC may provide the proliferative drive. These experiments shed new light on the process of acquired resistance to antiestrogens, implicating ELF5 as a potential therapeutic target in antiestrogen-resistant disease and providing a potential marker predicting the failure of antiestrogen therapy.

Overall we show that the transcriptional activity of ELF5 suppresses estrogen action in luminal breast cancer, enhances expression of the basal phenotype, specifies patterns of gene expression distinguishing molecular subtype, and exerts a proliferative influence that can be modified to allow luminal breast cancer to become resistant to antiestrogen treatment.

## Materials and Methods

### Ethics Statement

Mice were maintained following the Australian code of practice for the care and use of animals for scientific purposes observed by The Garvan Institute of Medical Research/St. Vincent's Hospital Animal Ethics Committee (AEC).

### Plasmid Construction, Transfection, Infection, and Treatment

ESE2B was tagged at the 3′ end with V5 and incorporated into the pHUSH-ProEX vector [35] used as a retrovirus. Transient transfection used FuGENE (Roche). Puromycin was used at 1 µg/ml for MCF7-EcoR and 2 µg/ml for T47D-EcoR, DOX at 0.1 µg/ml changed daily, R5020 at 10 nM.

### Colony Formation Assays

Colonies were visualized with the Diff Quick Stain Kit (Lab Aids) or crystal violet. Colony number and size measured with Image J 1.41o (Wayne Rasband, US National Institutes of Health), excluding cells <0.1 mm.

### Protein Analysis and Antibodies

Protein lysates (40 µg per lane) were prepared in NuPAGE LDS Sample Buffer and Sample Reducing Agent and separated on NuPAGE Bis-Tris acrylamide gels in MOPS buffer or Tris-Acetate gels run in Tris-Acetate SDS buffer, transferred to polyvinylidene difluoride membranes (BioRad), blocked with 1% skim milk powder, 50 nM Na_3_PO_4_, 50 mM NaCl, and 0.1% Tween 20 for 1 h, primary antibody for 2 h to overnight, and a horseradish peroxidase (HRP)-linked secondary antibody for 1 h, with four washes of 50 nM Na_3_PO_4_, 50 mM NaCl, and 0.1% Tween 20. Detection by enhanced chemiluminescence (Perkin-Elmer) on Fuji Medical X-ray Film (Fujifilm). Primary antibodies used were anti-β-actin (AC-15, Sigma), anti-cyclin B1 (GNS-11, BD), anti-cyclin D1 (DCS6, Novacastra), anti-cyclin E (HE12, sc-247, Santa Cruz), anti-ELF5/ESE2 (N20, sc-9645, Santa Cruz), anti**-**ERα (HC20, sc-543, Santa Cruz), anti-FAK (3285, Cell Signalling Technologies), anti-ILK (3/ILK, BD), anti-integrin β1 (18, BD), anti-p21 (70, BD), anti-p27 (C19, sc-528, Santa Cruz), anti-p107 (C19, sc-318, Santa Cruz), anti-pFAK (pY397, BD), anti-pRb (554136, BD), anti SRC (Calbiochem 327), anti pSRC (Cell Signalling 2101), and anti-V5 (R960-25, Invitrogen). Secondary antibodies were HRP-donkey anti-goat (Santa Cruz), HRP-donkey anti-rabbit (Amersham Biosciences), and HRP-sheep anti-mouse (Amersham Biosciences).

### Reporter Assays

Reporter plasmids, consensus ERE were a kind gift from Malcolm Parker (Imperial Cancer Research Fund, London); FOXA1 responsive UGT2B17/-155ApGL3 a kind gift from Peter Mackenzie (Flinders University, Australia) [36]; and pRL-TK (Promega), β-galactosidase a kind gift from Gerald Clesham, University of Cambridge, Cambridge, UK. Solubilization was in Passive Lysis Buffer (Promega) and measured with Dual Luciferase Reporter Assay System (Promega) and Galacto-Light reagents (Applied Biosystems).

### Attachment Assays

Monolayers were detached with 0.25% trypsin, counted, and re-plated at equal density. Unattached cells were gently removed in PBS prior to counting with a haemocytometer.

### Flow Cytometry

Asychronous cells were pulsed with 10 µM BrdU (Sigma) for 2 h (MCF7 or 20 min (T47D) prior to harvesting. Where indicated cells were synchronised with 1 mM hydroxyurea treatment for 40 h. Cells were harvested via trypsinisation, and fixed in 70% ethanol for 24 h, stained with 10 µg/ml propidium iodide (Sigma) for 2–5 h, and incubated with 0.5 mg/ml RNase A (Sigma). Flow cytometry was performed using FACS Calibur or FACS Canto cytometers (BD Biosciences), and data analysis performed with FlowJo software (Tree Star Inc.).

### Microarray Analysis

Total RNA was extracted with the RNeasy Minikit (Qiagen) and DNase-treated with the DNase kit (Qiagen). RNA quality was assessed using a RNA Nano LabChip and Agilent Bioanalyzer 2100. Probe labeling and hybridization to Affymetrix Human Gene 1.0 ST Gene Arrays was done at the Ramaciotti Centre for Gene Function Analysis at the University of New South Wales. Microarrays were normalised using RMA via the *NormalizeAffymetrixST* GenePattern module http://pwbc.garvan.unsw.edu.au/gp. Differentially expressed genes were detected using Limma, using positive false discovery rate (FDR) multiple hypothesis test correction. GSEA (version 2.04) used 1,000 permutations, in pre-ranked mode, using the t*-*statistic from Limma to rank each gene using gene sets from MSigDB version 3.0. Data are available from GEO with accession number GSE30407.

### ChIP-Sequencing Analysis

Cells were fixed in 1% formaldehyde, at 37°C for 10 min, washed 2× with cold PBS, scraped into 600 µl PBS with protease inhibitors (P8340, Sigma), spun 2 min at 6,000 *g*, washed as before, and snap frozen in liquid nitrogen. ChIP-Seq as previously described [37] using a 50%–50% mixture of V5-specific antibody and the Santa Cruz N20 anti ELF5 antibody. DNA was processed for Illumina sequencing using 36-bp reads on a GAIIx. Sequences were aligned against NCBI Build 36.3 of the human genome using MAQ (http://maq.sourceforge.net/) with default parameters. The aligned reads were converted to BED format using a custom script. ChIP-qPCR used the ABI Prism 7900HT Sequence Detection System.

### Mice

Balb/C Nude mice (Jackson Lab) were used as xenograft hosts.

### Breast Cancer Subtype

Potential as classifiers was investigated using the diagonal linear discriminant analysis (DLDA) or Naïve Bayes classifier, 100 iterations of 10-fold cross-validation (CV) and the misclassification rate recorded in each instance. Boxplots of ER misclassification rate were achieved for each of the 1,000 (100 iterations of 10-fold CV) classifiers built to predict ER status. The confusion matrix shows as percentages the relationship between true intrinsic subtype classification, as defined by [1] and [38] and predicted by the Naïve Bayes classifier. Expression signature analysis [15] and GSEA [30] to examine changes in subtype was carried out as described.

## Supporting Information

Figure S1 ***ELF5*** **expression in the breast cancer molecular subtypes.** Top panels. Tumors from the indicated studies were classified by molecular subtype and their *ELF5* expression level given by the Affymetrix probe set 220625_s_at is graphed. Similar results were found with the 220624_s_at probes (not shown) The *p*-value for differential expression of *ELF5* by the basal subtype is shown. Bottom panel, Redrawn from Ma et al. [21] to combine the clinical data given as text with the graphical format. Ma et al. used laser capture microdissection to analyse *ELF5* expression in tumor and patient-associated adjacent normal epithelium. Data are expressed as fold change in expression and is colored according to ER and PR status. Grade 3 tumors are indicated by asterisks.
(TIF)

Figure S2 ***ELF5*** **expression in breast cancer.** Each row shows the results from a study referenced by Oncomine. Total numbers of tumors are indicated including type, lobular (LBC), or ductal (DBC). Cell color indicates average fold change (see scale) in *ELF5* expression for the indicated phenotypic comparison contained within each cell. *p*-Values are given where <0.05.
(TIF)

Figure S3 **Construction and validation of an inducible** ***ELF5-V5*** **expression system in MCF7 and T47D breast cancer cell lines.** (A) the retroviral expression vector was constructed as indicated using the Genentech pHUSH ProEX vector. Addition of DOX relives repression of cytomeglovirus promoter (CMV)-driven expression of *ELF5* tagged by V5, by binding the tetracycline repressor (TetR) and removing it from the *Tet* operon (TO). TetR expression is linked to Puromycin resistance (*PURO*) via an internal ribosome entry site (*IRES*) ensuring coexpression of these activities. Control cells were constructed using this vector without the *ELF5-V5* cassette. (B) Quantification of *ELF5* isoforms in T47D and MCF7 cells. qPCR specific to each isoform was used to compare expression levels. Amplification efficiency was very similar for both assays. Left-hand side panel, *ESE2B* was expressed at levels more than three orders of magnitude greater than *ESE2A*. Right-hand panel shows relative *ESE2A* expression. (C) Induction of *ELF5-V5* expression increases the mRNA level of its direct transcriptional target, whey acidic protein (*Wap*) compared to an empty vector control plasmid, in HC11 mouse mammary epithelial cells. Cells were treated from day 4 by the lactogenic hormones prolactin, insulin and hydrocortisone.
(TIF)

Figure S4 **Visualization of the transcriptional functions of ELF5 in breast cancer.** GSEA-identified signatures indicative of function within expression profiles derived from forced *ELF5* expression in T47D and MCF7 luminal breast cancer cells. Results are visualized using the enrichment map plug-in for Cytoscape. Each node is a gene set, diameter indicates size, outer node color represents the magnitude and direction of enrichment (see scale) in T47D cells, inner node color enrichment in MCF7 cells. Thickness of the edges (green lines) is proportional the similarity of linked nodes. The most related clusters are placed nearest to each other. View the PDF at 800% or 1,600% to explore the network in detail.
(PDF)

Figure S5 **Visualization of the transcriptional functions of ELF5 in breast cancer cell cycle and cancer gene set sub network in T47D and MCF7 cells.** Inset, the region of the complete network from Figure S4 that is highlighted in red and yellow is expanded here. Main panel, nodes, and edges forming the cell cycle and cancer-related network, as explained in the key and legend of Figure S4.
(TIF)

Figure S6 **Visualization of the transcriptional functions of ELF5 in breast cancer molecular subtype network in T47D and MCF7 cells.** Inset, the region of the complete network from Figure S4 highlighted in red and yellow is expanded here. Main panel, nodes, and edges forming the molecular subtype network as explained in the key and legend of Figure S4.
(TIF)

Figure S7 **Heat maps of leading edge genes contained within the cell cycle cluster in T47D cells.** Gene expression is high (dark red), middle (white), or low (purple) in row- normalized depictions of gene expression levels. First two columns from the left are duplicates –DOX then next two are duplicates +DOX. Labels indicate gene set name.
(TIF)

Figure S8 **Heat maps of leading edge genes contained within the Cytoscape cancer cluster in T47D cells.** Gene expression is high (dark red), middle (white), or low (purple) in row-normalized depictions of expression levels. First two columns from the left are duplicates –DOX then next two are duplicates +DOX. Labels indicate gene set name.
(TIF)

Figure S9 **Heat maps of leading edge genes contained within the Cytoscape breast cancer subtype cluster in T47D or MCF7 cells.** Gene expression is high (dark red), middle (white), or low (purple) in row-normalized depictions of expression levels. First two columns from the left are duplicates –DOX then next two are duplicates +DOX. Labels indicate cell model and gene set name.
(TIF)

Figure S10 **Heat maps of leading edge genes contained within the Cytoscape breast cancer estrogen response cluster in T47D cells.** Gene expression is high (dark red), middle (white), or low (purple) in row-normalized depictions of expression levels. First two columns from the left are duplicates –DOX then next two are duplicates +DOX. Labels indicate gene set name.
(TIF)

Figure S11 **Visualization of the transcriptional functions of ELF5 in luminal A breast cancers.** GSEA identified signatures indicative of function within an expression profile derived by correlation of gene expression with *ELF5* expression in luminal A breast cancers from the UNC337 series using Pearson correlation. Results are visualized using the enrichment map plug-in for Cytoscape. Each node is a gene set, diameter indicates size, node color represents the magnitude and direction of enrichment. Thickness of the edges (green lines) is proportional to the similarity of linked nodes. The most related clusters are placed nearest to each other. View the PDF at 800% or 1,600% to explore the network in detail.
(PDF)

Figure S12 **ELF5 modulates breast cancer cell accumulation.** (A) Effects of DOX addition on cell number at 0 h (+D 0 h, closed circles), or at 24 h (+D24 h, closed circles), or at 48 h (+D48 h, closed circles), or not added (−D, open circles). Inset, comparison of the level of exogenous ELF5-V5 induction by DOX with that effected by treatment with the progestin R5020 on endogenous ELF5. (B) Effect of DOX withdrawal on cell number. Cells carrying the *ELF5-V*5 cassette were plated with DOX (+D0 h, closed circles) and remained on DOX or were withdrawn from DOX (+D0 h −D24 h, open circles) after 24 h. Inset decay in ELF5-V5 expression by Western blot. (C) T47D-ELF5-V5 cells were grown with (+D) or without (−D) DOX on agar gels for 3 wk. Colony numbers from pooled cells or a clonal line are shown. (D) T47D (circles, dashed lines) or MCF7 cells (squares, solid lines) were transfected with siRNA against *ELF5* mRNA (siELF5, solid symbols), or a RISC-complex inactive control siRNA (risc, open symbols). Inset, The degree of *ELF5* mRNA knockdown was measured by qPCR at 72 h. (E and F) quantification of Western blots in Figure 5D, showing the effects of ELF5-V5 induction on cell adhesion molecules.
(TIF)

Figure S13 **ELF5 modulates cell proliferation.** (A) T47D-V5 and MCF7-V5 cells were treated with DOX for 48 h. DNA was labeled by BrdU incorporation for 2 h and analysed by flow cytometry using propidium iodide to measure total DNA content. BrdU incorporation (*y* axis) and DNA content (*x* axis) distinguish G0–G1, S, and G2-M phases of the cell cycle, with phase distribution expressed as a percentage of total cells (*p<0.05, **p<0.005). (B) Changes in the expression of the indicated key cell cycle regulatory genes with time measured by Western blot. (C) Changes in the expression of the indicated cell cycle regulatory genes with time measured by qPCR. (D) Flow cytometric profiles of hydroxyl urea arrested cells released into cycle, from which the data in Figure 4H were derived. (E) Quantification of Figure 4F, changes in cell cycle regulatory proteins occurring following the release of hydroxyl urea arrested cells.
(TIF)

Figure S14 **Expression of the ELF5 transcriptional signature in breast cancer.** (A) hypergeometric interaction between 943 genes repressed (DN) or 966 genes induced (UP) by forced expression of ELF5-V5 in T47D cells, or 785 ELF5 ChIP targets in T47D, with a 641 gene proliferation signature. (B) Left-hand side heat map shows the expression change in response to the induction of ELF5-V5 in T47D cells of the 55 ELF5 ChIP targets involved in proliferation that were identified in (A). Right-hand side heat map shows the expression of the 55 ELF5 ChIp targets in the Desmedt breast cancer series. *p*-Values and fold change (FC) for genes is shown where significant differential expression was observed between ER− and ER+ cancers. (C) Breast cancer series in Oncomine showing significant enrichment of the 55 ELF5 ChIP targets. (D) Heat maps showing the expression change of estrogen induced (E2 I) or repressed (E2 R) genes in response to forced ELF5-V5 expression (+D). Annotations at the side are examples of enriched gene sets with p values. T = T47D, M = MCF7. (E) Heat maps illustrating examples of differential expression of the ELF5 ChIP targets in relation to prognostic indicators within some of the breast cancer series indicated in Figure 5E by asterisks.
(TIF)

Figure S15 **The ELF5 transcriptional signature correctly distinguishes breast cancer subtype.** An ELF5 transcriptional signature was defined as ELF5 ChIP targets with robust changes in expression in response to forced ELF5 expression in T47D cells. It was used to predict ER status (A) or breast cancer subtype (B) in the Reyal series. Rows of the confusion matrix show percent correct subtype prediction at the shaded cells and the distribution of confused predictions by subtype across the row. (C) Ability of this ELF5 transcriptional signature to predict breast cancer subtype and clinical characteristics in the NKI295 series. The 55 Elf5 gene signature was used to cluster the NKI295 series. Subtypes assigned to this series by its authors are colored as indicated. Heat map shows gene expression levels with enriched gene sets within the major gene clusters listed along side. Bottom panel shows associated clinical correlates with the significance of each colored bar indicated by the text at the right. Generally good outcomes are in yellow, poor outcomes in purple or red.
(TIF)

Figure S16 **Visualization of the transcriptional functions of ELF5 in breast and mammary cancer.** GSEA-identified signatures indicative of function within expression profiles derived from forced *ELF5* expression in T47D luminal breast cancer cells and knockdown of ELF5 function in HCC1937 basal breast cancer cells. Results are visualized using the enrichment map plug-in for Cytoscape. Each node is a gene set, diameter indicates size, outer node color represents the magnitude and direction of enrichment (see scale) in HCC1937 cells, inner node color enrichment in T47D cells. Thickness of the edges (green lines) is proportional the similarity of linked nodes. The most related clusters are placed nearest to each other. The functions of prominent clusters are shown.
(TIF)

Figure S17 **ELF5 specifies breast cancer subtype.** GSEA network derived from forced ELF5 expression in T47D luminal breast cancer cells (inner node color) and knockdown of ELF5 expression in HCC1937 basal breast cancer cells (outer node color). Node size is proportional to gene set size, thicker green lines indicate greater leading edge gene overlap. Nodes are positioned according to similarity in leading edge genes. Labels indicate the functional significance of the four clusters generated.
(TIF)

Table S1 **ELF5 ChIP targets and expression changes in T47D-ELF5-V5 cells.** Spreadsheet showing the ELF5 ChIP targets and their changed levels of expression in response to forced ELF5-V5 expression in T47D cells.
(XLSX)

## Abbreviations

ChIP-Seq: chromatin immunoprecipitation of DNA followed by DNA sequencing
DOX: doxycycline
ER: estrogen receptor
ESE: epithelium specific
FAK: focal adhesion kinase
FASR: Faslodex
G1: gap 1 of the cell cycle
GSEA: gene set enrichment analysis
PR: progesterone receptor
qPCR: quantitative PCR
TAMR: Tamoxifen
TSS: transcription start site
