# Supplementary figures and images for: ELF5 Suppresses Estrogen Sensitivity and Underpins the Acquisition of Antiestrogen Resistance in Luminal Breast Cancer
Source: PLoS Biol. 2012 Dec 27;10(12):e1001461. doi: 10.1371/journal.pbio.1001461 (PMC3531499; doi:10.1371/journal.pbio.1001461)

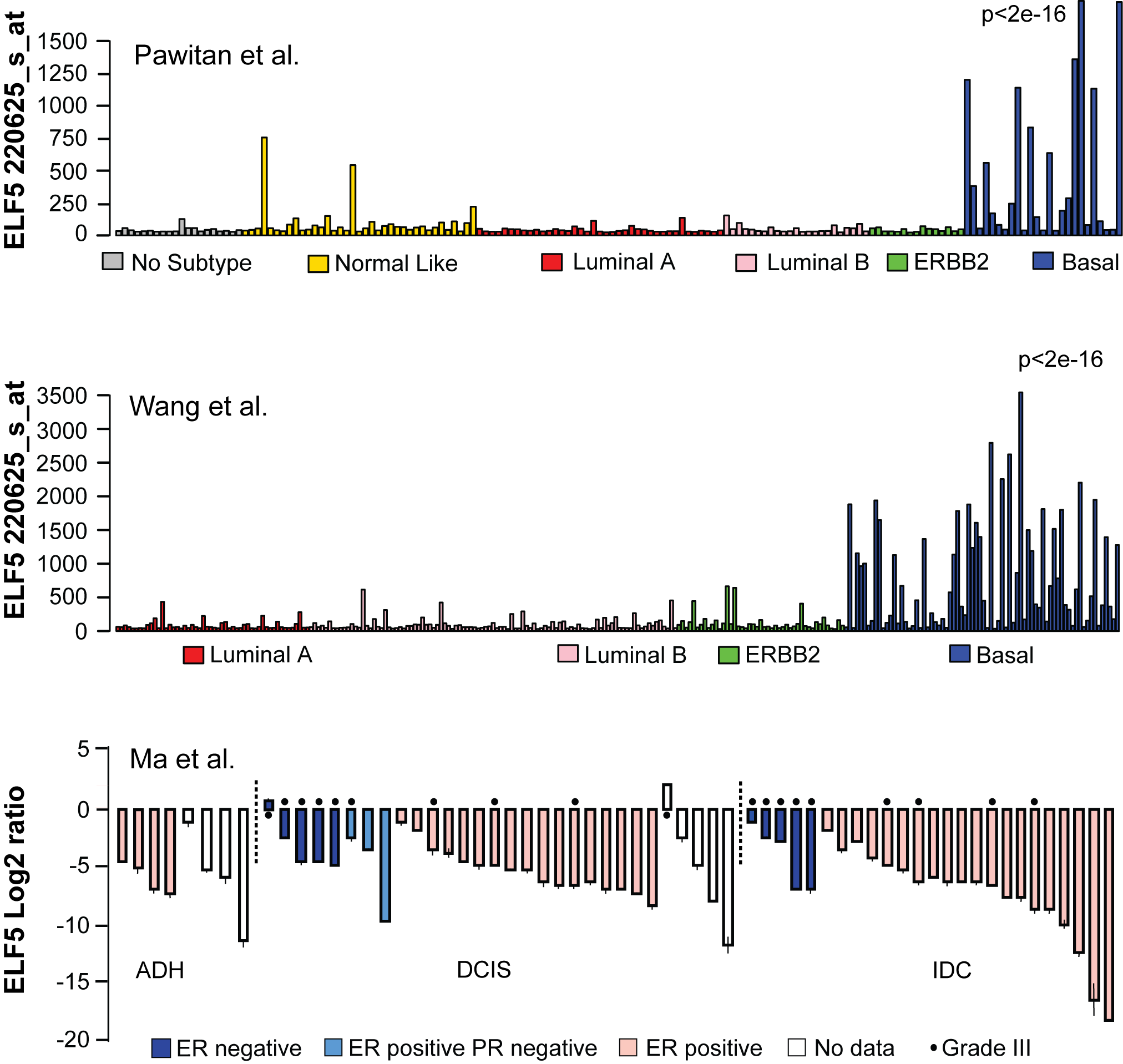

Supplement: Figure S1 — ELF5 expression in the breast cancer molecular subtypes. Top panels. Tumors from the indicated studies were classified by molecular subtype and their ELF5 expression level given by the Affymetrix probe set 220625_s_at is graphed. Similar results were found with the 220624_s_at probes (not shown) The p-value for differential expression of ELF5 by the basal subtype is shown. Bottom panel, Redrawn from Ma et al. [21] to combine the clinical data given as text with the graphical format. Ma et al. used laser capture microdissection to analyse ELF5 expression in tumor and patient-associated adjacent normal epithelium. Data are expressed as fold change in expression and is colored according to ER and PR status. Grade 3 tumors are indicated by asterisks. (TIF) [file pbio.1001461.s001.tif]

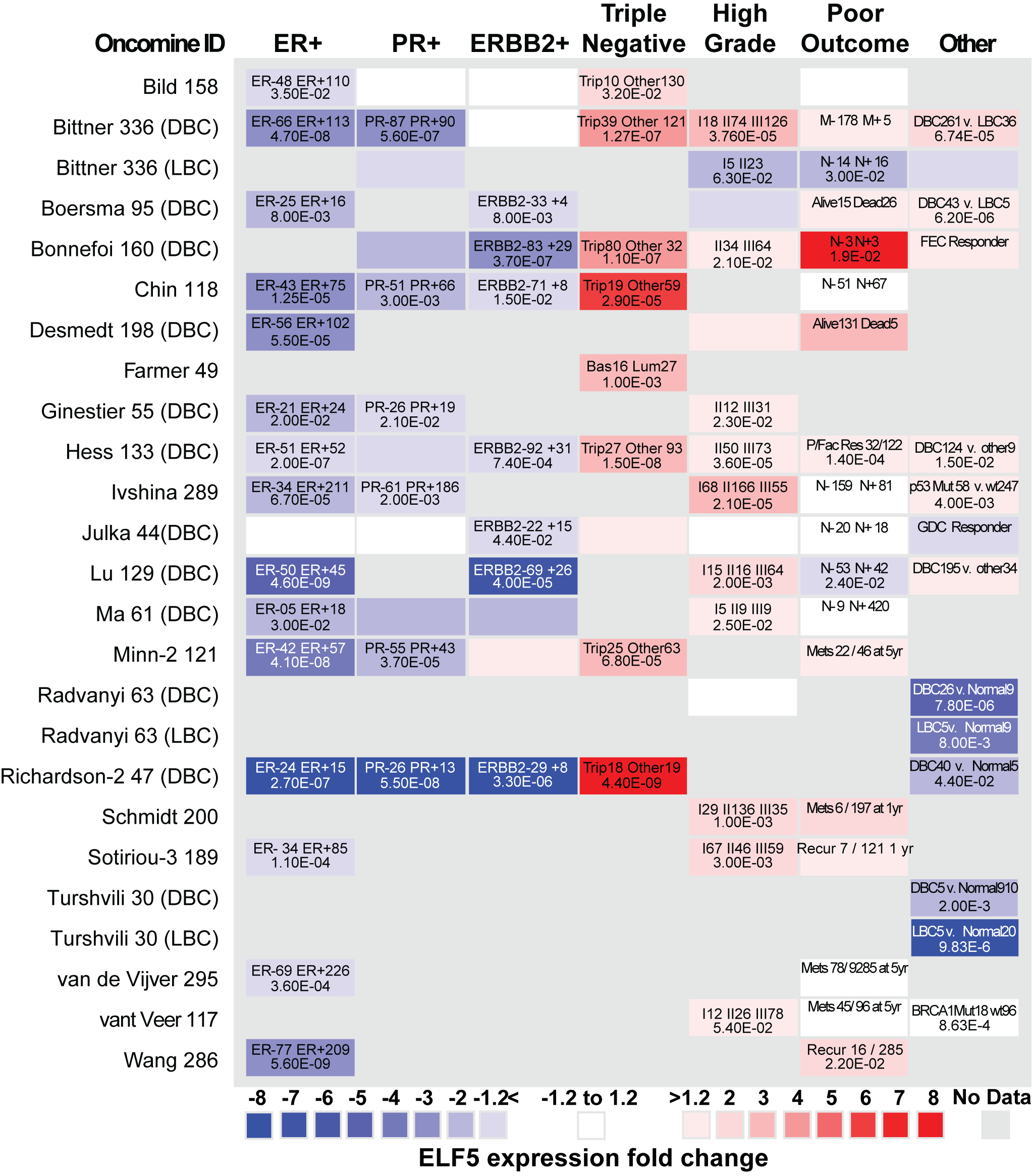

Supplement: Figure S2 — ELF5 expression in breast cancer. Each row shows the results from a study referenced by Oncomine. Total numbers of tumors are indicated including type, lobular (LBC), or ductal (DBC). Cell color indicates average fold change (see scale) in ELF5 expression for the indicated phenotypic comparison contained within each cell. p-Values are given where <0.05. (TIF) [file pbio.1001461.s002.tif]

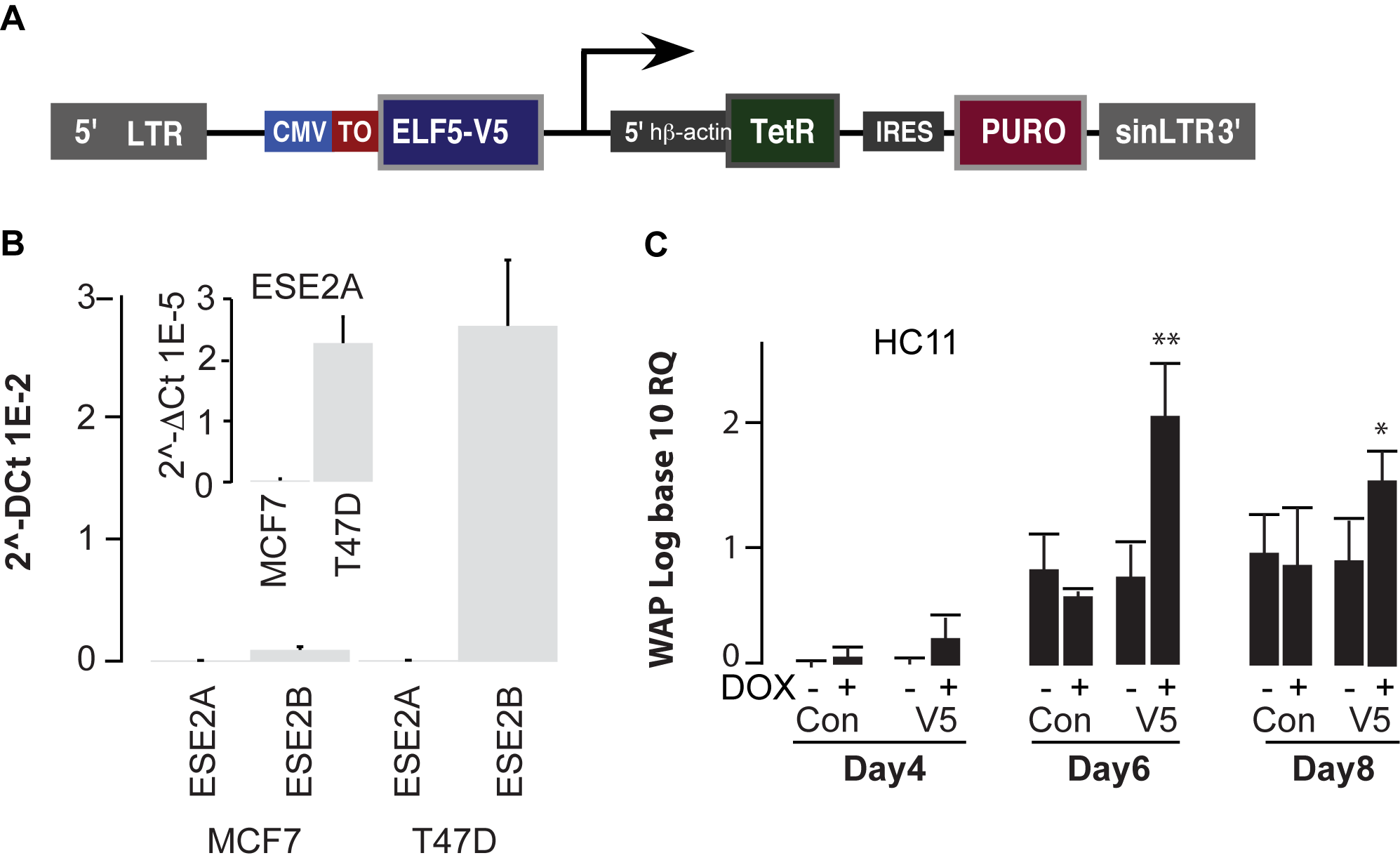

Supplement: Figure S3 — Construction and validation of an inducible ELF5-V5 expression system in MCF7 and T47D breast cancer cell lines. (A) the retroviral expression vector was constructed as indicated using the Genentech pHUSH ProEX vector. Addition of DOX relives repression of cytomeglovirus promoter (CMV)-driven expression of ELF5 tagged by V5, by binding the tetracycline repressor (TetR) and removing it from the Tet operon (TO). TetR expression is linked to Puromycin resistance (PURO) via an internal ribosome entry site (IRES) ensuring coexpression of these activities. Control cells were constructed using this vector without the ELF5-V5 cassette. (B) Quantification of ELF5 isoforms in T47D and MCF7 cells. qPCR specific to each isoform was used to compare expression levels. Amplification efficiency was very similar for both assays. Left-hand side panel, ESE2B was expressed at levels more than three orders of magnitude greater than ESE2A. Right-hand panel shows relative ESE2A expression. (C) Induction of ELF5-V5 expression increases the mRNA level of its direct transcriptional target, whey acidic protein (Wap) compared to an empty vector control plasmid, in HC11 mouse mammary epithelial cells. Cells were treated from day 4 by the lactogenic hormones prolactin, insulin and hydrocortisone. (TIF) [file pbio.1001461.s003.tif]

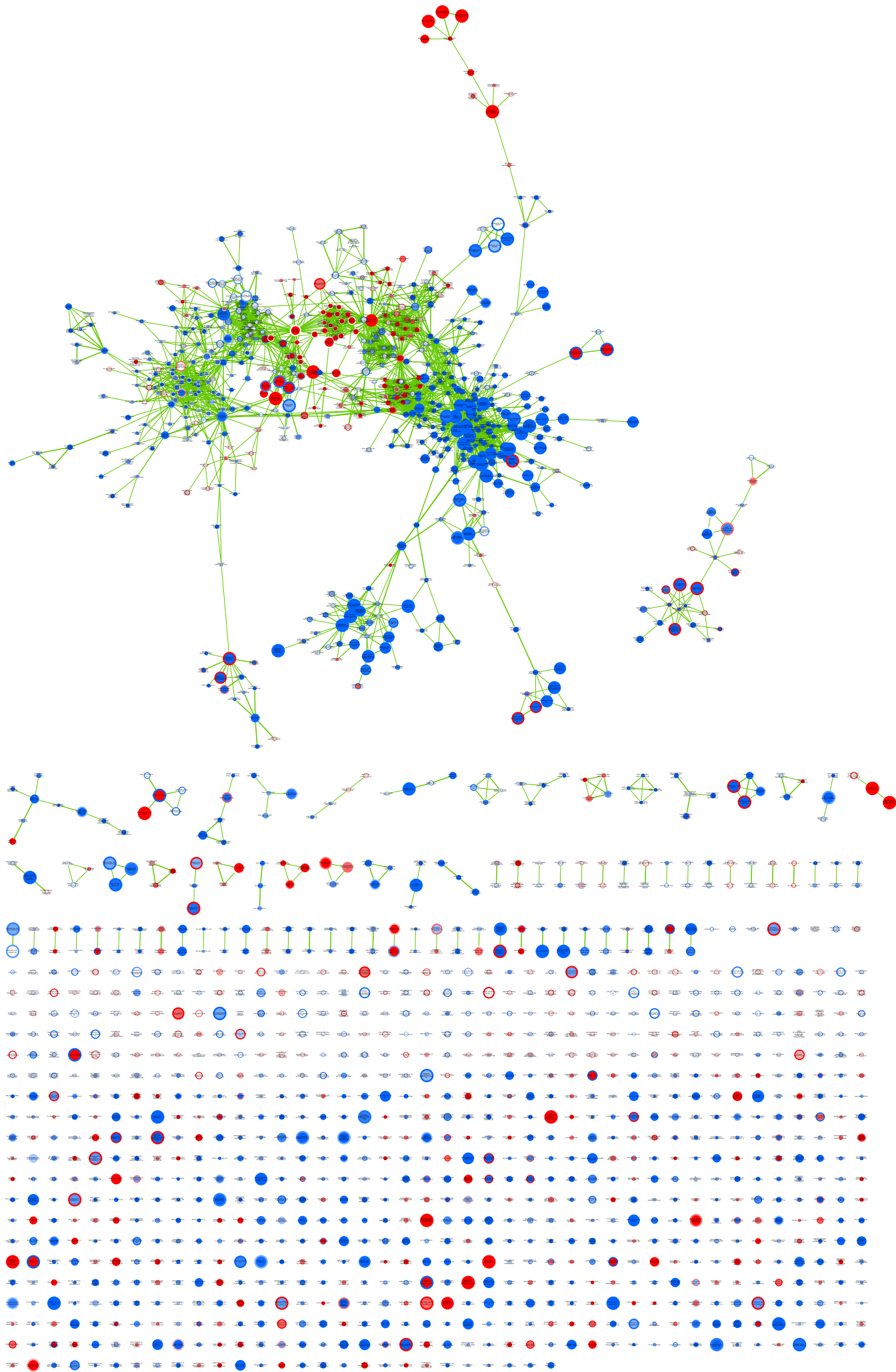

Supplement: Figure S4 — Visualization of the transcriptional functions of ELF5 in breast cancer. GSEA-identified signatures indicative of function within expression profiles derived from forced ELF5 expression in T47D and MCF7 luminal breast cancer cells. Results are visualized using the enrichment map plug-in for Cytoscape. Each node is a gene set, diameter indicates size, outer node color represents the magnitude and direction of enrichment (see scale) in T47D cells, inner node color enrichment in MCF7 cells. Thickness of the edges (green lines) is proportional the similarity of linked nodes. The most related clusters are placed nearest to each other. View the PDF at 800% or 1,600% to explore the network in detail. (PDF) [file pbio.1001461.s004.pdf]

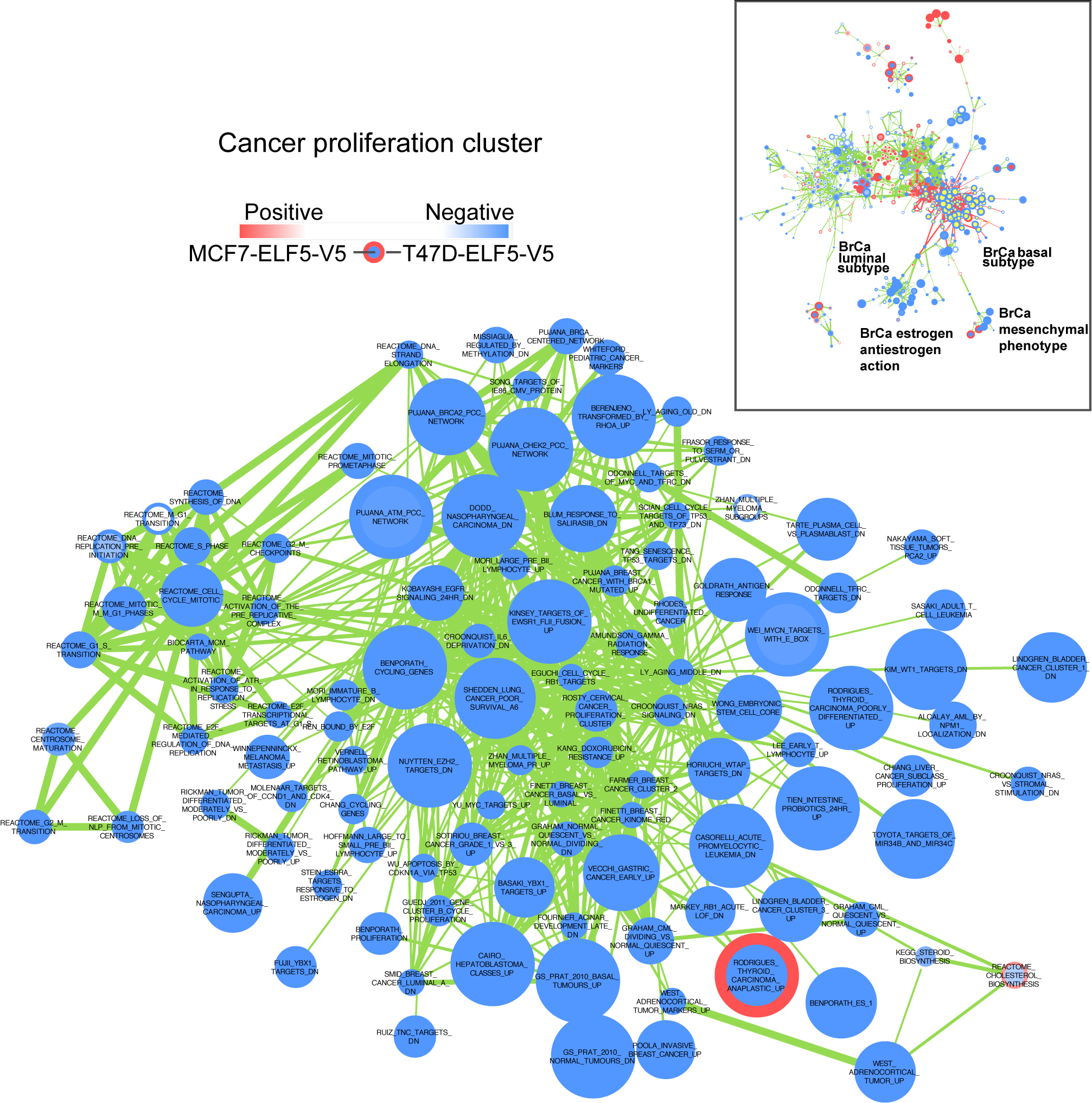

Supplement: Figure S5 — Visualization of the transcriptional functions of ELF5 in breast cancer cell cycle and cancer gene set sub network in T47D and MCF7 cells. Inset, the region of the complete network from Figure S4 that is highlighted in red and yellow is expanded here. Main panel, nodes, and edges forming the cell cycle and cancer-related network, as explained in the key and legend of Figure S4. (TIF) [file pbio.1001461.s005.tif]

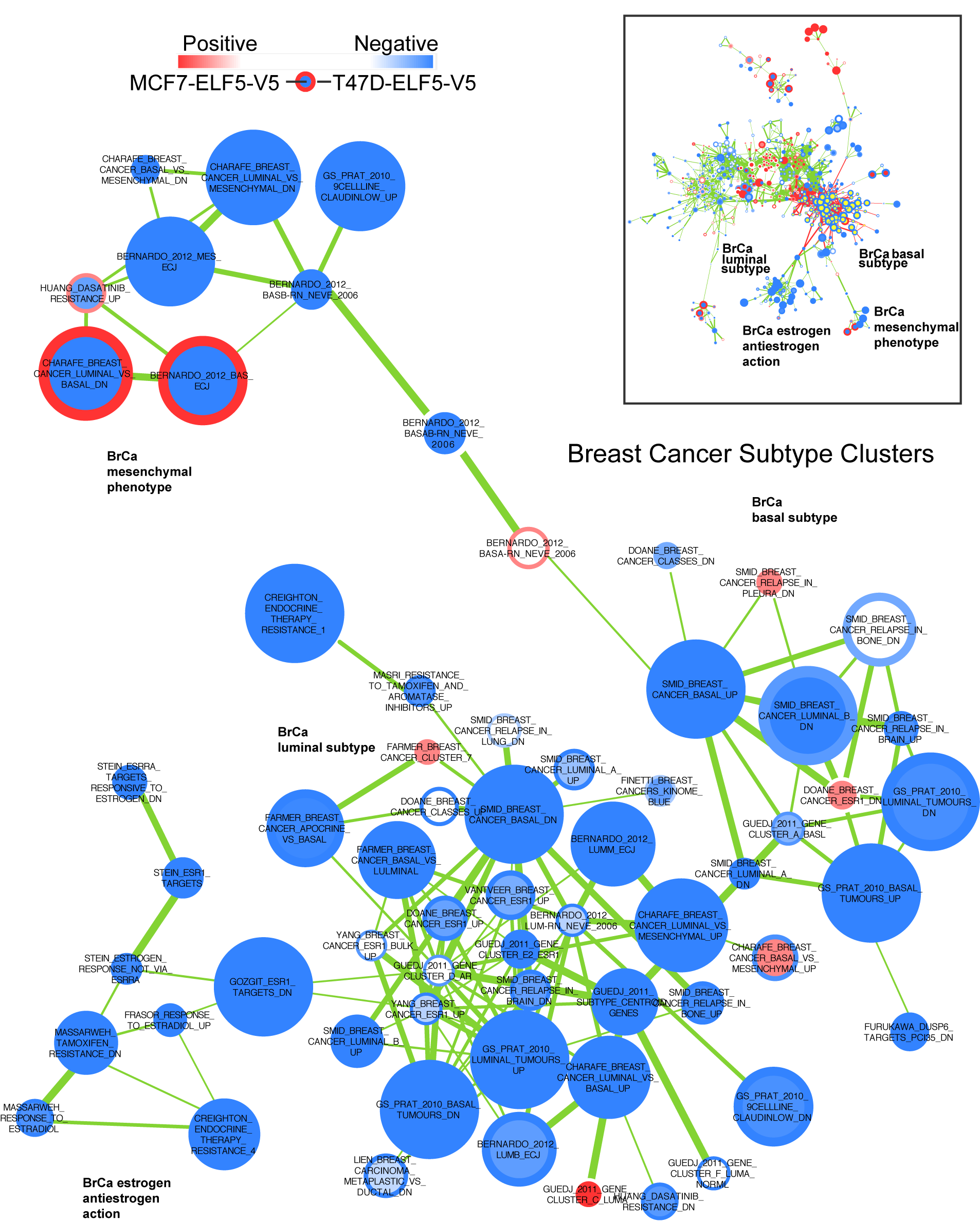

Supplement: Figure S6 — Visualization of the transcriptional functions of ELF5 in breast cancer molecular subtype network in T47D and MCF7 cells. Inset, the region of the complete network from Figure S4 highlighted in red and yellow is expanded here. Main panel, nodes, and edges forming the molecular subtype network as explained in the key and legend of Figure S4. (TIF) [file pbio.1001461.s006.tif]

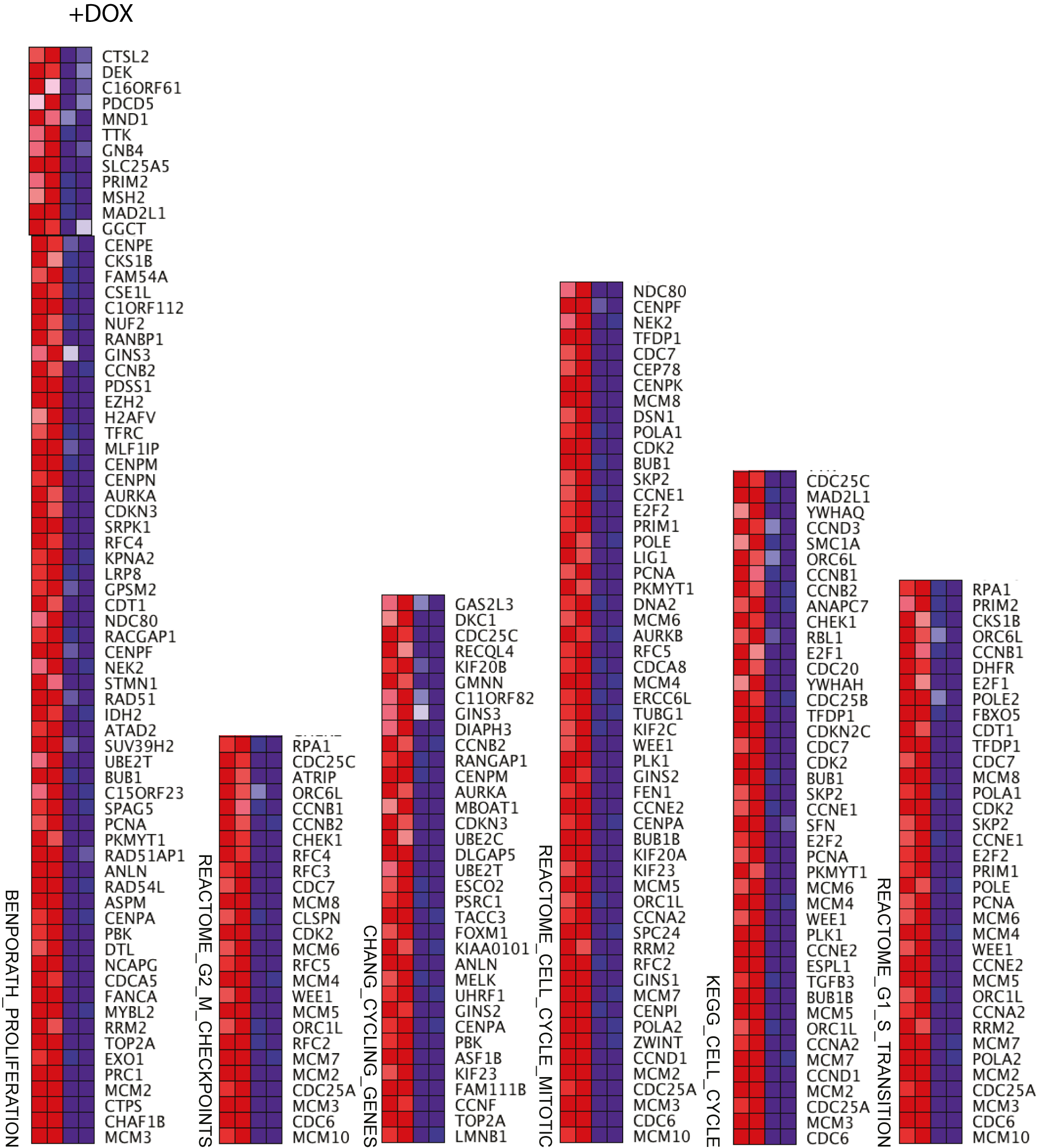

Supplement: Figure S7 — Heat maps of leading edge genes contained within the cell cycle cluster in T47D cells. Gene expression is high (dark red), middle (white), or low (purple) in row- normalized depictions of gene expression levels. First two columns from the left are duplicates –DOX then next two are duplicates +DOX. Labels indicate gene set name. (TIF) [file pbio.1001461.s007.tif]

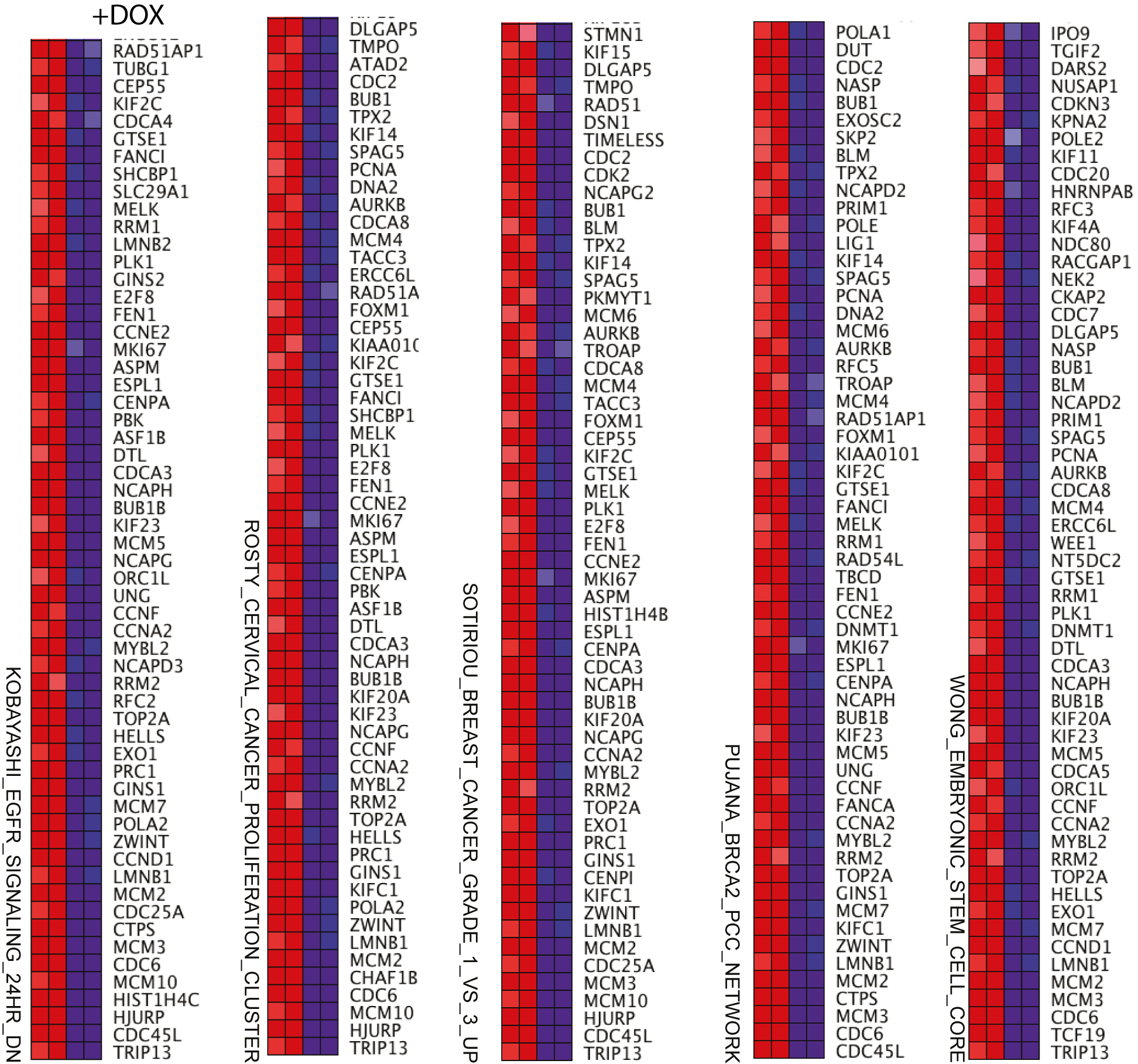

Supplement: Figure S8 — Heat maps of leading edge genes contained within the Cytoscape cancer cluster in T47D cells. Gene expression is high (dark red), middle (white), or low (purple) in row-normalized depictions of expression levels. First two columns from the left are duplicates –DOX then next two are duplicates +DOX. Labels indicate gene set name. (TIF) [file pbio.1001461.s008.tif]

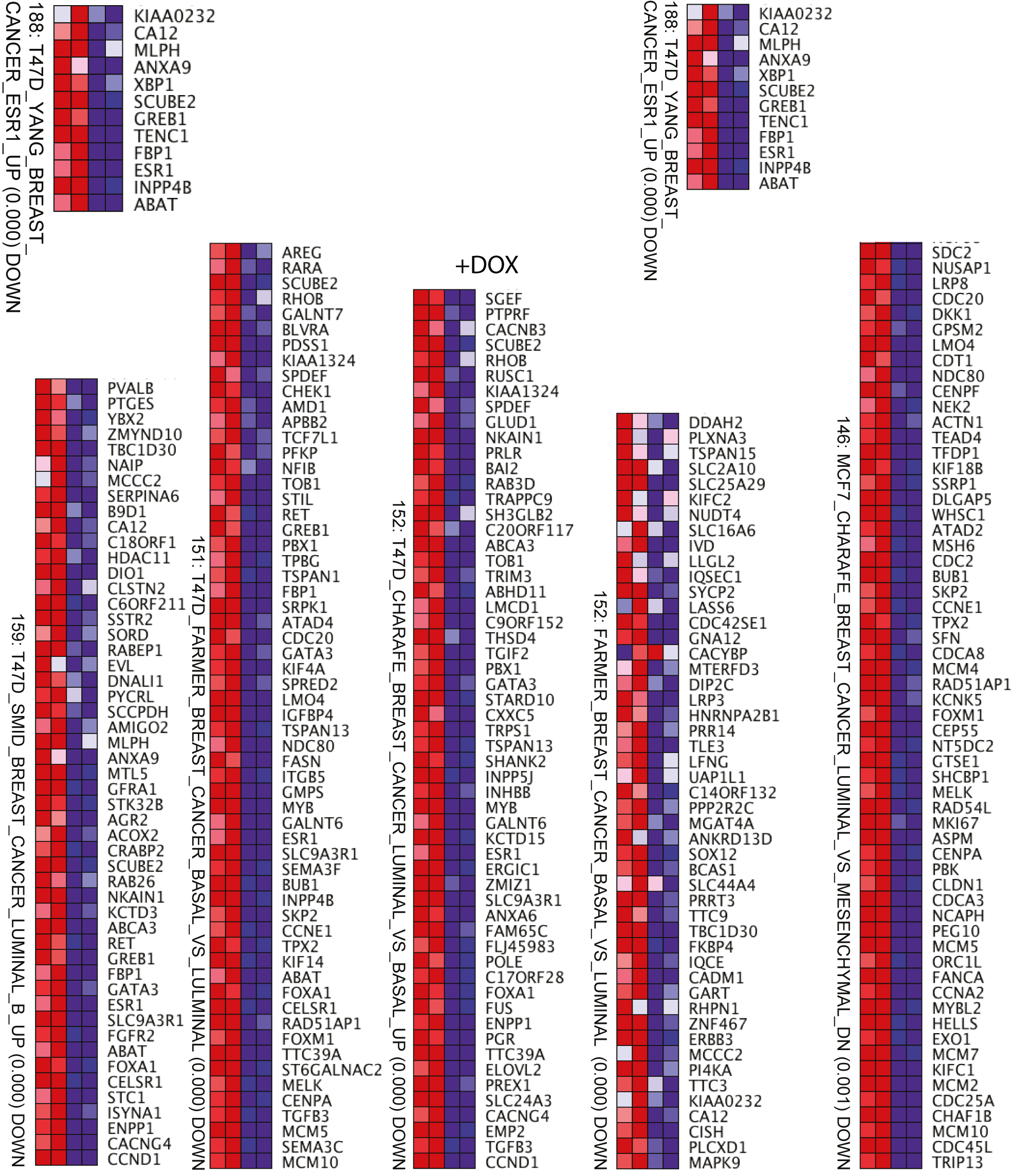

Supplement: Figure S9 — Heat maps of leading edge genes contained within the Cytoscape breast cancer subtype cluster in T47D or MCF7 cells. Gene expression is high (dark red), middle (white), or low (purple) in row-normalized depictions of expression levels. First two columns from the left are duplicates –DOX then next two are duplicates +DOX. Labels indicate cell model and gene set name. (TIF) [file pbio.1001461.s009.tif]

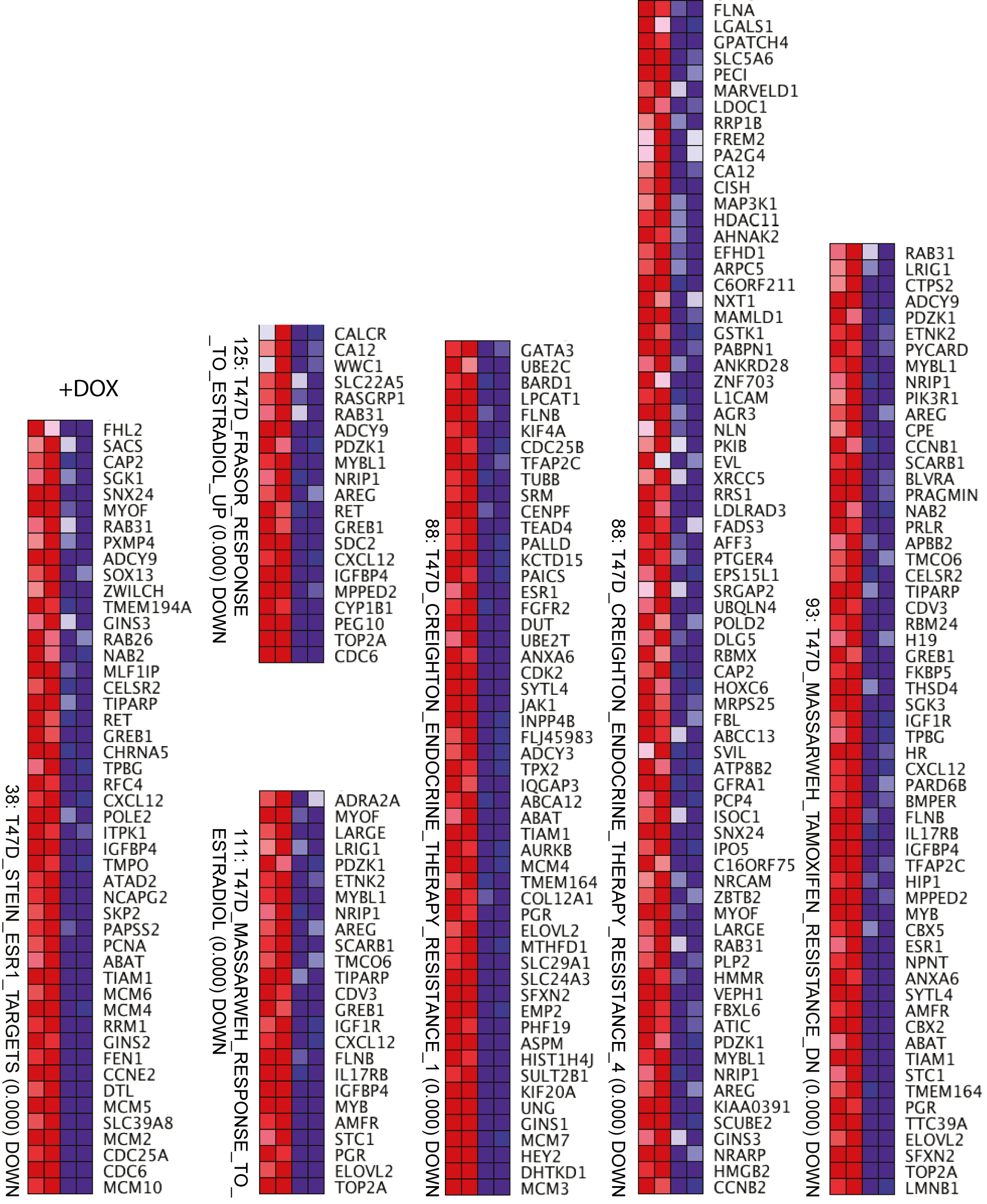

Supplement: Figure S10 — Heat maps of leading edge genes contained within the Cytoscape breast cancer estrogen response cluster in T47D cells. Gene expression is high (dark red), middle (white), or low (purple) in row-normalized depictions of expression levels. First two columns from the left are duplicates –DOX then next two are duplicates +DOX. Labels indicate gene set name. (TIF) [file pbio.1001461.s010.tif]

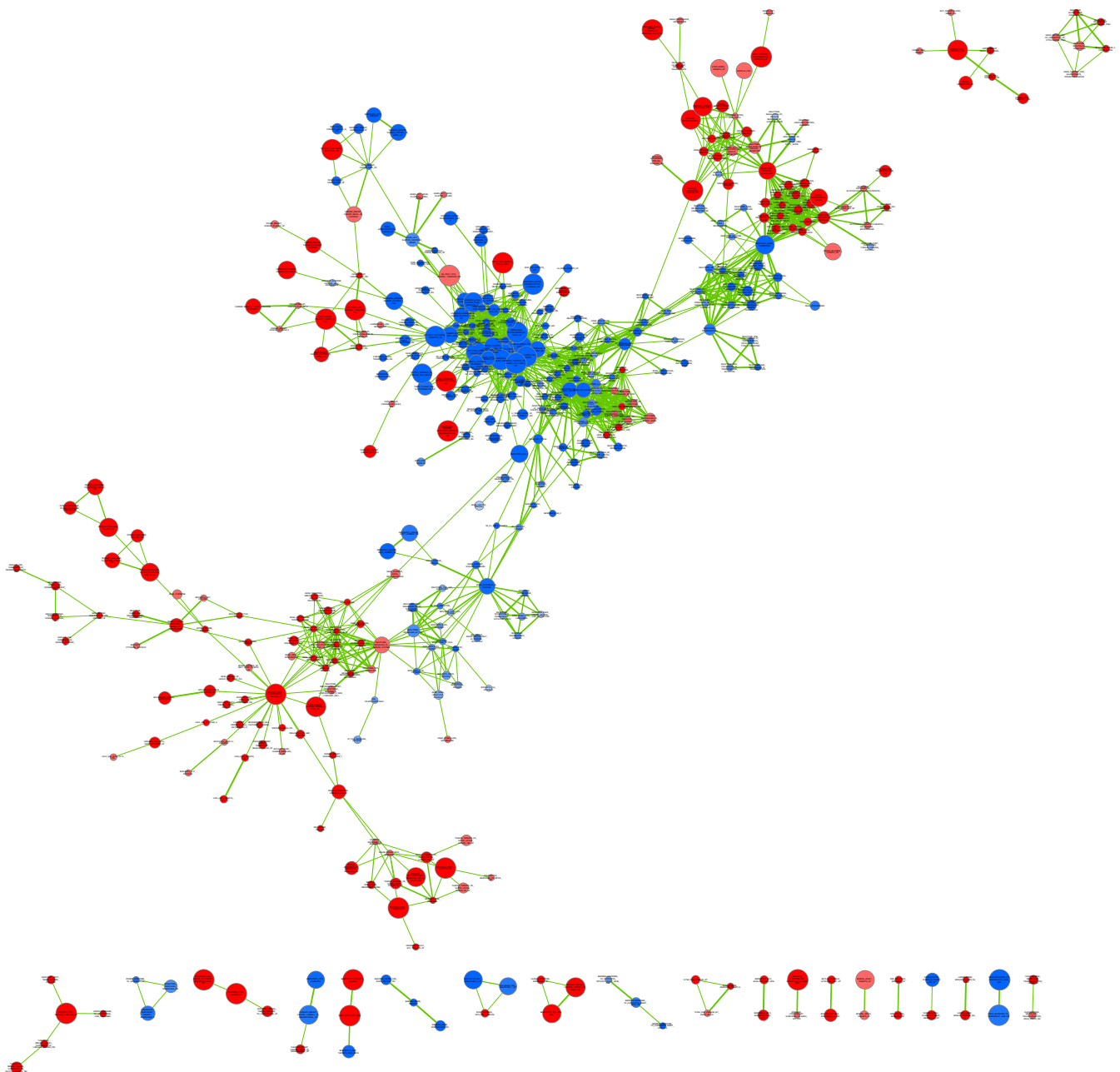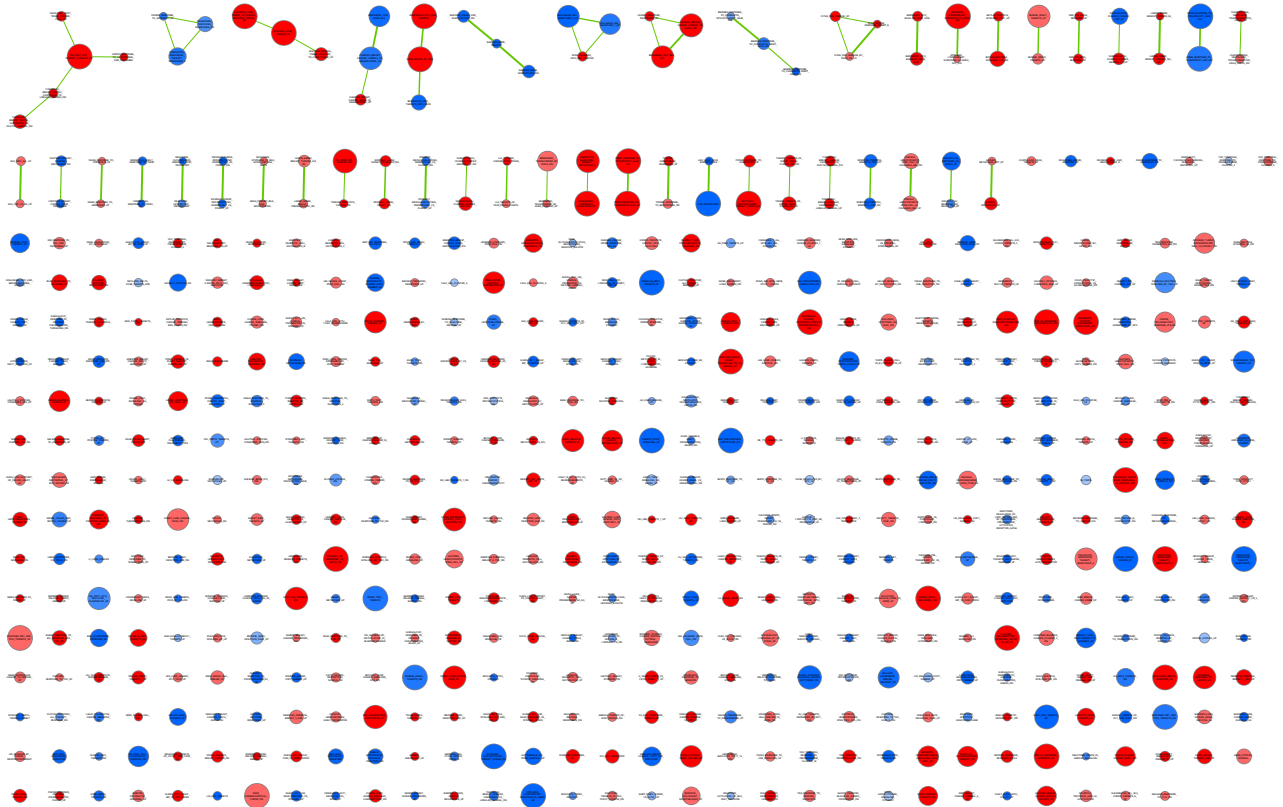

Supplement: Figure S11 — Visualization of the transcriptional functions of ELF5 in luminal A breast cancers. GSEA identified signatures indicative of function within an expression profile derived by correlation of gene expression with ELF5 expression in luminal A breast cancers from the UNC337 series using Pearson correlation. Results are visualized using the enrichment map plug-in for Cytoscape. Each node is a gene set, diameter indicates size, node color represents the magnitude and direction of enrichment. Thickness of the edges (green lines) is proportional to the similarity of linked nodes. The most related clusters are placed nearest to each other. View the PDF at 800% or 1,600% to explore the network in detail. (PDF) [file pbio.1001461.s011.pdf]

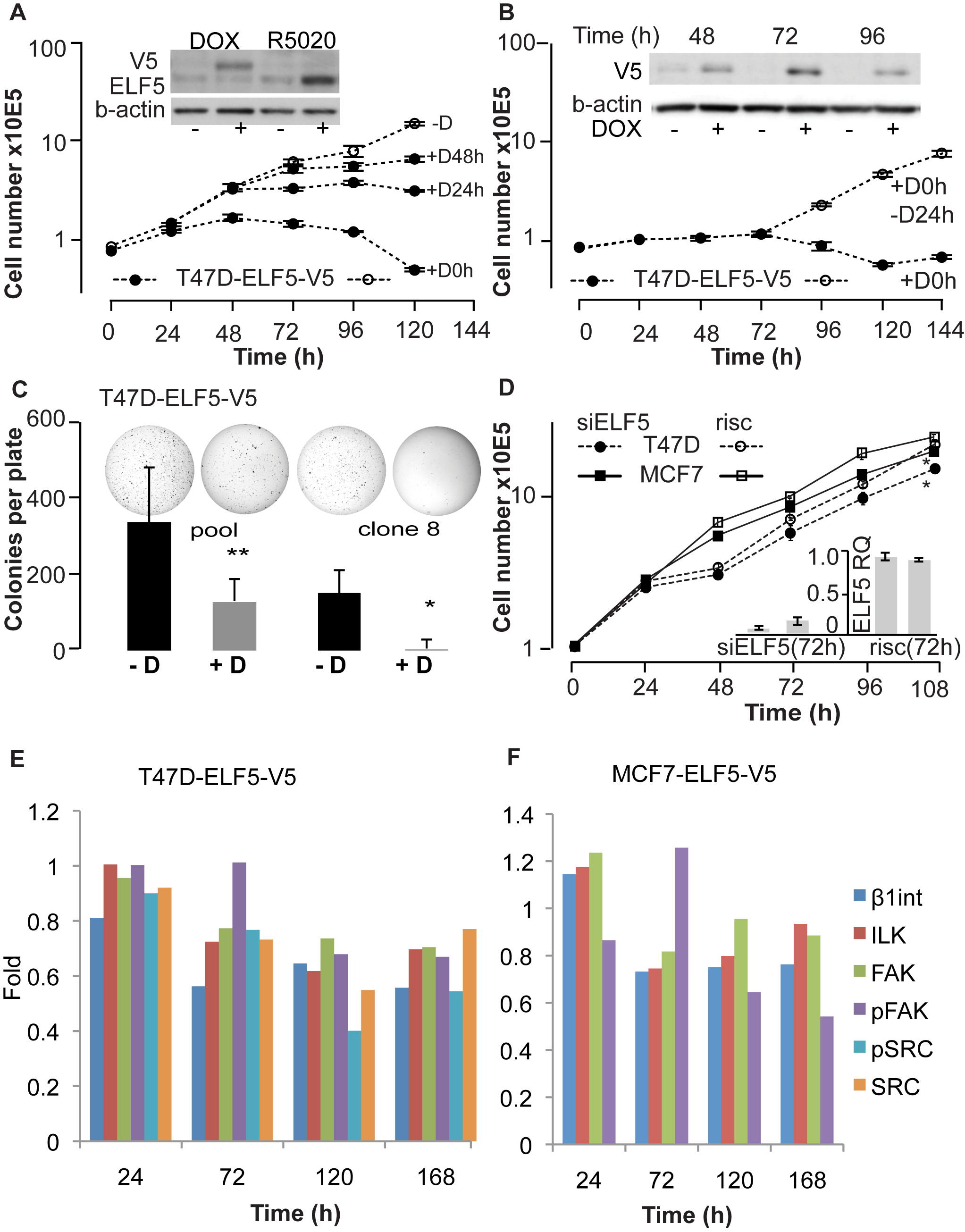

Supplement: Figure S12 — ELF5 modulates breast cancer cell accumulation. (A) Effects of DOX addition on cell number at 0 h (+D 0 h, closed circles), or at 24 h (+D24 h, closed circles), or at 48 h (+D48 h, closed circles), or not added (−D, open circles). Inset, comparison of the level of exogenous ELF5-V5 induction by DOX with that effected by treatment with the progestin R5020 on endogenous ELF5. (B) Effect of DOX withdrawal on cell number. Cells carrying the ELF5-V5 cassette were plated with DOX (+D0 h, closed circles) and remained on DOX or were withdrawn from DOX (+D0 h −D24 h, open circles) after 24 h. Inset decay in ELF5-V5 expression by Western blot. (C) T47D-ELF5-V5 cells were grown with (+D) or without (−D) DOX on agar gels for 3 wk. Colony numbers from pooled cells or a clonal line are shown. (D) T47D (circles, dashed lines) or MCF7 cells (squares, solid lines) were transfected with siRNA against ELF5 mRNA (siELF5, solid symbols), or a RISC-complex inactive control siRNA (risc, open symbols). Inset, The degree of ELF5 mRNA knockdown was measured by qPCR at 72 h. (E and F) quantification of Western blots in Figure 5D, showing the effects of ELF5-V5 induction on cell adhesion molecules. (TIF) [file pbio.1001461.s012.tif]

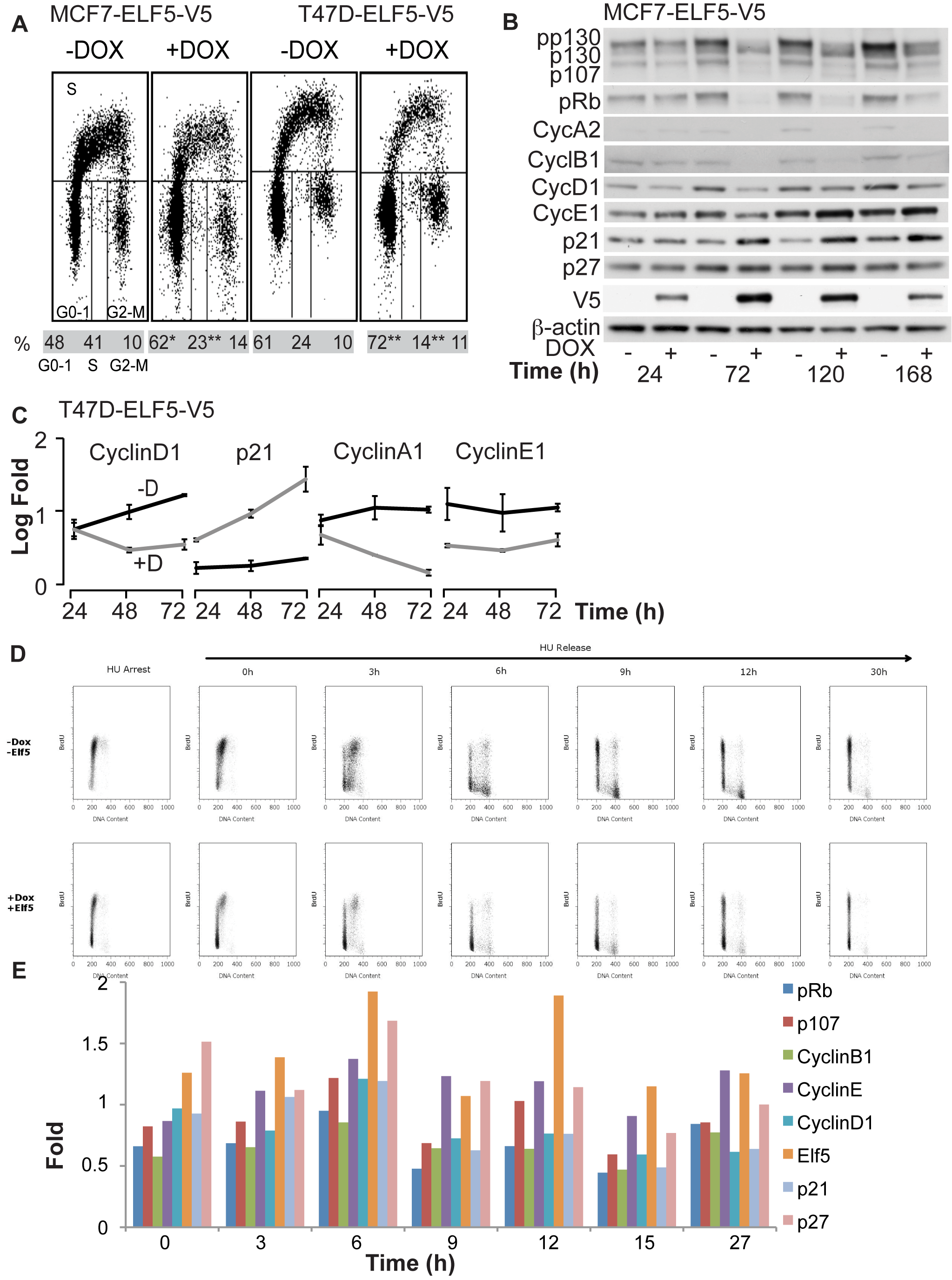

Supplement: Figure S13 — ELF5 modulates cell proliferation. (A) T47D-V5 and MCF7-V5 cells were treated with DOX for 48 h. DNA was labeled by BrdU incorporation for 2 h and analysed by flow cytometry using propidium iodide to measure total DNA content. BrdU incorporation (y axis) and DNA content (x axis) distinguish G0–G1, S, and G2-M phases of the cell cycle, with phase distribution expressed as a percentage of total cells (*p<0.05, **p<0.005). (B) Changes in the expression of the indicated key cell cycle regulatory genes with time measured by Western blot. (C) Changes in the expression of the indicated cell cycle regulatory genes with time measured by qPCR. (D) Flow cytometric profiles of hydroxyl urea arrested cells released into cycle, from which the data in Figure 4H were derived. (E) Quantification of Figure 4F, changes in cell cycle regulatory proteins occurring following the release of hydroxyl urea arrested cells. (TIF) [file pbio.1001461.s013.tif]

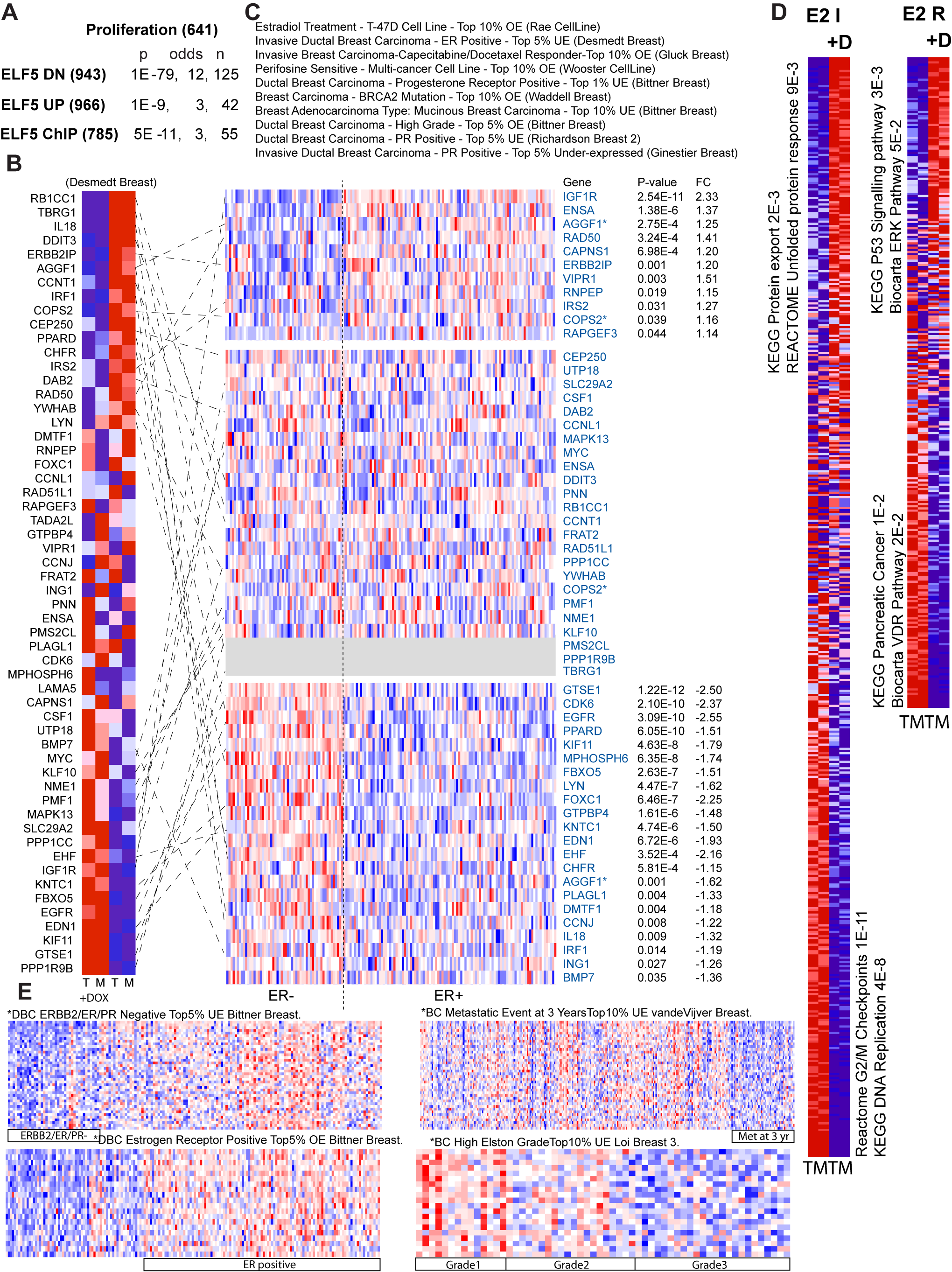

Supplement: Figure S14 — Expression of the ELF5 transcriptional signature in breast cancer. (A) hypergeometric interaction between 943 genes repressed (DN) or 966 genes induced (UP) by forced expression of ELF5-V5 in T47D cells, or 785 ELF5 ChIP targets in T47D, with a 641 gene proliferation signature. (B) Left-hand side heat map shows the expression change in response to the induction of ELF5-V5 in T47D cells of the 55 ELF5 ChIP targets involved in proliferation that were identified in (A). Right-hand side heat map shows the expression of the 55 ELF5 ChIp targets in the Desmedt breast cancer series. p-Values and fold change (FC) for genes is shown where significant differential expression was observed between ER− and ER+ cancers. (C) Breast cancer series in Oncomine showing significant enrichment of the 55 ELF5 ChIP targets. (D) Heat maps showing the expression change of estrogen induced (E2 I) or repressed (E2 R) genes in response to forced ELF5-V5 expression (+D). Annotations at the side are examples of enriched gene sets with p values. T = T47D, M = MCF7. (E) Heat maps illustrating examples of differential expression of the ELF5 ChIP targets in relation to prognostic indicators within some of the breast cancer series indicated in Figure 5E by asterisks. (TIF) [file pbio.1001461.s014.tif]

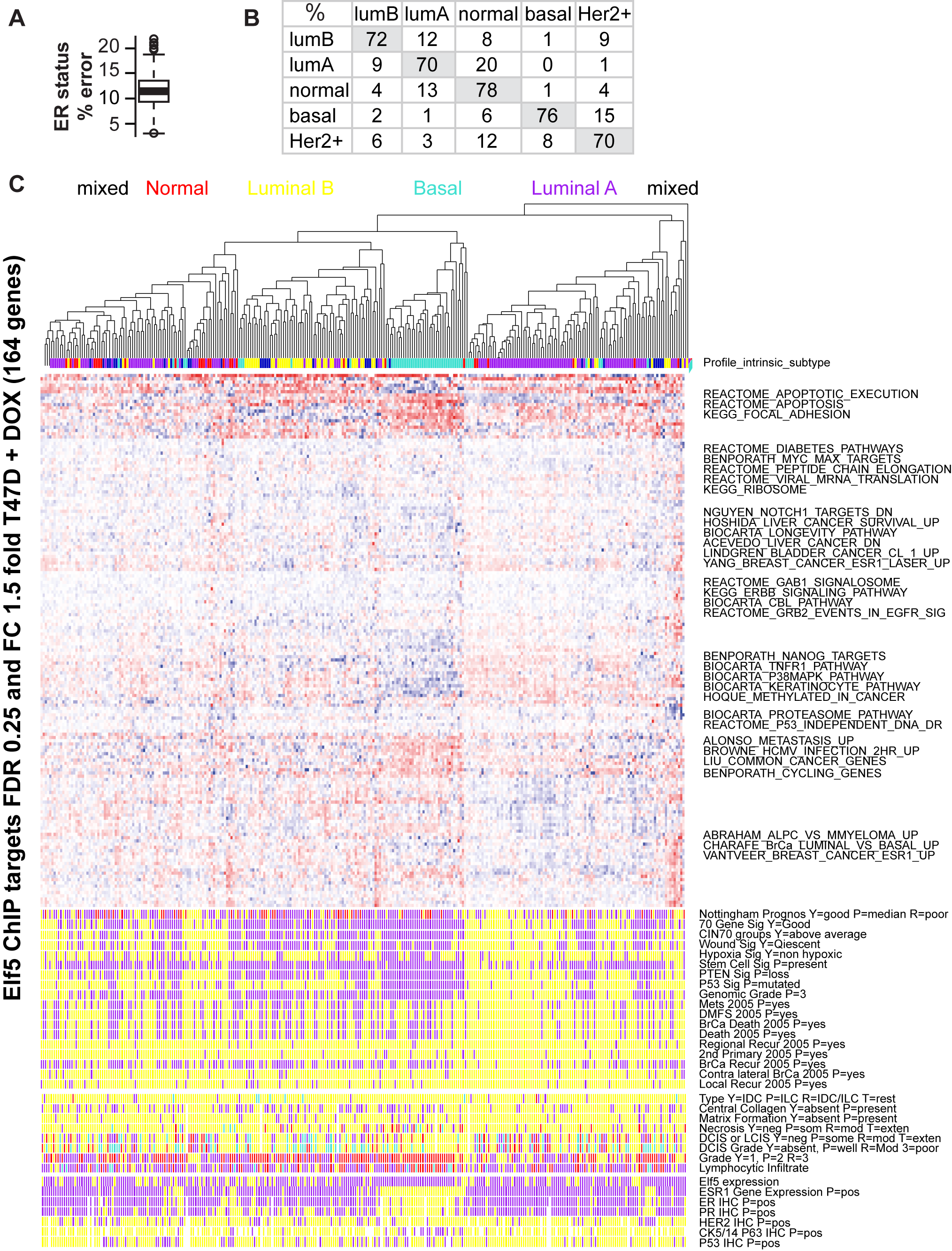

Supplement: Figure S15 — The ELF5 transcriptional signature correctly distinguishes breast cancer subtype. An ELF5 transcriptional signature was defined as ELF5 ChIP targets with robust changes in expression in response to forced ELF5 expression in T47D cells. It was used to predict ER status (A) or breast cancer subtype (B) in the Reyal series. Rows of the confusion matrix show percent correct subtype prediction at the shaded cells and the distribution of confused predictions by subtype across the row. (C) Ability of this ELF5 transcriptional signature to predict breast cancer subtype and clinical characteristics in the NKI295 series. The 55 Elf5 gene signature was used to cluster the NKI295 series. Subtypes assigned to this series by its authors are colored as indicated. Heat map shows gene expression levels with enriched gene sets within the major gene clusters listed along side. Bottom panel shows associated clinical correlates with the significance of each colored bar indicated by the text at the right. Generally good outcomes are in yellow, poor outcomes in purple or red. (TIF) [file pbio.1001461.s015.tif]

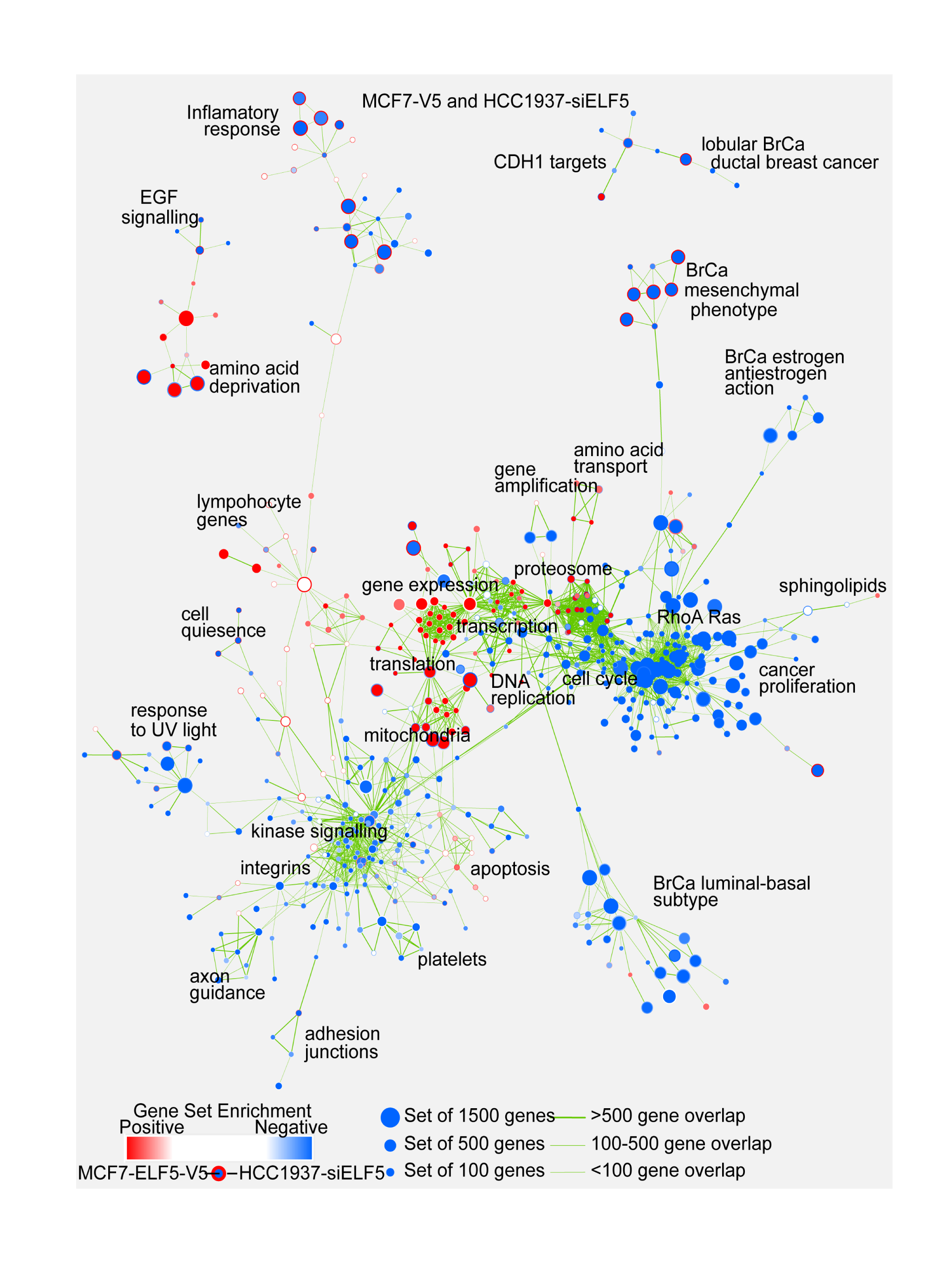

Supplement: Figure S16 — Visualization of the transcriptional functions of ELF5 in breast and mammary cancer. GSEA-identified signatures indicative of function within expression profiles derived from forced ELF5 expression in T47D luminal breast cancer cells and knockdown of ELF5 function in HCC1937 basal breast cancer cells. Results are visualized using the enrichment map plug-in for Cytoscape. Each node is a gene set, diameter indicates size, outer node color represents the magnitude and direction of enrichment (see scale) in HCC1937 cells, inner node color enrichment in T47D cells. Thickness of the edges (green lines) is proportional the similarity of linked nodes. The most related clusters are placed nearest to each other. The functions of prominent clusters are shown. (TIF) [file pbio.1001461.s016.tif]

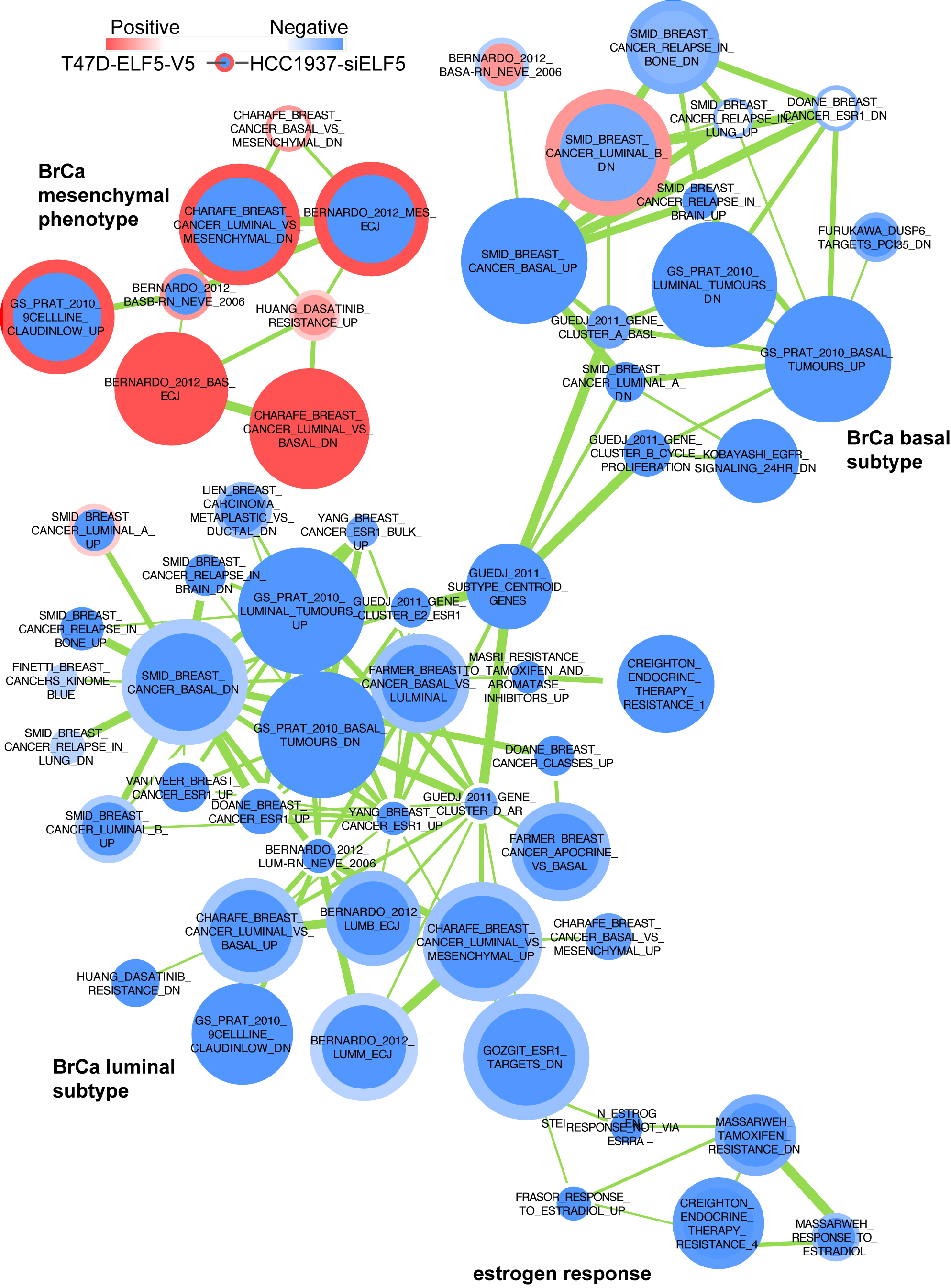

Supplement: Figure S17 — ELF5 specifies breast cancer subtype. GSEA network derived from forced ELF5 expression in T47D luminal breast cancer cells (inner node color) and knockdown of ELF5 expression in HCC1937 basal breast cancer cells (outer node color). Node size is proportional to gene set size, thicker green lines indicate greater leading edge gene overlap. Nodes are positioned according to similarity in leading edge genes. Labels indicate the functional significance of the four clusters generated. (TIF) [file pbio.1001461.s017.tif]
